# DMS‐MaPseq and DREEM Analyses Implicate the Critical Role of RNA Structural Dynamics in Turnip Yellow Mosaic Virus Pathogenicity

**DOI:** 10.1002/advs.75614

**Published:** 2026-05-08

**Authors:** Jiaying Zhu, Changhao Li, Chisimbily Onyia, Xindi Li, Xingxing Yan, Songxiao Zhong, Taerin Oh, Xiuren Zhang

**Affiliations:** ^1^ Institute of Emerging Agricultural Technology Shenzhen University of Advanced Technology Shenzhen Guangdong China; ^2^ Department of Biochemistry and Biophysics Texas A&M University, College Station Texas USA; ^3^ Faculty of Synthetic Biology Shenzhen University of Advanced Technology Shenzhen Guangdong China

## Abstract

Increasing evidence indicates that RNA secondary structure (RSS) affects the life cycle of the RNA viruses in mammals. However, the specific roles of RSS in plant virus propagation are under‐studied. Here we decoded the in vivo genome‐wide RSS of Turnip Yellow Mosaic Virus (TYMV). We found that TYMV possesses a complicated RSS landscape across the genome in vivo. Furthermore, the viral RSS is dynamic in most parts of the genome. We discovered that the earlier reported pseudoknot on the 3’ tRNA‐like structure (3’ TLS) from in vitro assays indeed exists in vivo. Importantly, 3’ TLS also forms a novel alternative conformation that lacks the pseudoknot character, suggestive of the dynamics between two conformations. The 3’ TLS structural dynamics is critical for viral replication, whereas the pseudoknot structure is pivotal for translation. Thus, our findings characterized the in vivo genome‐wide RNA structural feature of TYMV and provided valuable insights for viral replication and translation in cells.

## Graphical abstract

RNA structural profiling of Turnip Yellow Mosaic Virus by DMS‐MaPseq and DREEM analyses uncover that viral genome‐wide RSS is highly complicated and heterogeneous, with alternative RSSs widely distributed across the genome. Notably, the viral 3’ tRNA‐like structure adopts alternative conformations in vivo. The structural dynamics play critical regulatory roles in viral translation and replication.

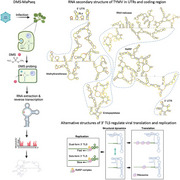

## 1 Introduction

The canonical function of RNA is to decode genetic information from DNA according to the central dogma. A growing number of studies in recent years have shown that RNA structure contains a new code of genetic information, as it has been implicated in transcript splicing, localization, turnover, translation, and so on [1]. RNA viruses utilize structural RNA motifs to co‐opt or manipulate host machinery and orchestrate viral processes such as translation or replication [2, 3]. A classic example is the internal ribosome entry sites (IRES), which can form 2D or 3D structures of viral RNAs to facilitate cap‐independent translation. More examples include the trinucleotide bulge and hexanucleotide loop on trans‐activation responsive region (TAR) RNA elements of HIV‐1 that are important for transcription [4, 5]. The development of chemical probing coupled with high‐throughput sequencing in the past decades has allowed the global survey of critical RSS in vivo [6, 7, 8, 9, 10, 11, 12, 13]. For example, genome‐wide RNA structure profiling of HIV‐1 [14] reveals a higher level of RNA structure in the spacer linkers between proteins as well as between protein domains. This pattern suggests the potential role of RNA structure in facilitating native folding of HIV proteins and functional domains by stalling ribosome elongation in the protein‐protein and domain‐domain junctions, given the earlier reports that highly‐structured RNA can cause ribosomal pausing [15, 16]. Besides, global RNA structure profiling of Zika virus (ZIKV) reveals an RNA interaction between 5’ UTR and E protein coding region that is critical to viral infection [17]. More recently, short‐ and long‐range interactions in the HIV‐1 5’ UTR are discovered to regulate viral genome dimerization and packing [18]. In addition, RNA structure mapping of influenza A virus revealed long‐range RNA interaction that is essential to virus genome packaging [19]. As RSS profiling techniques advance, an increasing number of regulatory RSSs have been identified, underscoring the growing need for a deeper understanding of RSS functionality.

The structure of macro biomolecules is often believed to be “wiggling and giggling” in vivo. Defining a single and favored structure for an RNA sequence over another one might be biologically biased and less meaningful. In addition to their sequence and structure, the motion and conformational alternations of RNA also play crucial roles in their activities and functions, particularly when a single RNA sequence performs multiple functions. This is exemplified by the structural heterogeneity observed at A3 splice sites in HIV‐1 RNA, where the alternative structures influence the generation of splicing products [20]. However, the study of RNA structural heterogeneity and structural function is still rudimentary, partially due to the techniques’ limitations. Consequently, the rules between alternative RNA structures and functions are largely obscure.

Numerous RNA viruses harbor tRNA‐like structures in their genomes. The tRNA mimicry is a powerful and elegant strategy for viruses to manipulate the host machinery and regulate essential processes, such as HIV‐1 [21], Israeli Acute Paralysis Virus (IAPV) [22, 23], and Cricket Paralysis Virus (CrPV) [24]. The 3’ TLS of *Tymovirus* and *Tobamovirus* are known to recruit specific host aminoacyl tRNA synthetases to aminoacylate the 3’ end of the viral RNA, which can further bind to eEF1A and ribosomes [25, 26]. Nonetheless, aminoacylation on 3’ TLS has been reported to be critical in both replication [27, 28, 29, 30] and translation [31, 32]. It raises a long‐term unresolved question of how the 3’ TLS ensures its recognition by either translation or replication machinery.

Turnip Yellow Mosaic Virus (TYMV) is characterized by a 6.3 kb single‐stranded positive‐sense RNA genome. It belongs to *Tymoviridae* family and infects a wide range of cruciferous plants such as turnips, radishes, and cabbage. The infected crops show yellowing leaves, stunted growth, and reduced yield, which cause economic loss and food security problems. The genome contains three open reading frames (ORFs), which produce 206K, 69K, and coating protein (CP). The 206K is a polyprotein, which is further self‐cleave into methyltransferase, peptidase, helicase, and RNA‐dependent RNA polymerase (RdRP). It has been long known that TYMV possesses a tRNA‐like structure at the 3’ end (3’ TLS) by in vitro X‐ray crystallography and NMR [26, 33]. The functional relevance of the structure remains unclear in a physiological context.

Here, we applied dimethyl sulfate mutational profiling with sequencing (DMS‐MaPseq) to profile the genome‐wide RSS landscape of TYMV in vivo. We found complicated RSS scenery across the TYMV genome. Interestingly, DREEM analysis (detection of RNA folding ensembles using expectation‐maximization) shows that genome‐wide RSS is highly dynamic and heterogeneous as alternative RSSs are prevalent across the genome including the 5’ UTR and the famous 3’ TLS region. We then focused on 3’ TLS and indeed observed the in vivo presence of the pseudoknot structure on the 3’ TLS, reminiscent of its in vitro conformation previously reported. Intriguingly, we have also uncovered a novel alternative conformation of the 3' TLS (minor structure) in vivo. Importantly, mutations on the pseudoknot structure (major structure) compromised the translation, whereas the mutations on either major or minor structures blocked the viral replication, indicating the pivotal role of structural dynamics in viral replication. Thus, this study not only provided a comprehensive map of genome‐wide RSS and structural diversity for a plant RNA virus, but also underscored the significance of structural dynamics to RNA multifunctionality.

## 2 Results

### 2.1 DMS‐MaPseq Depicts RSS Landscape of TYMV In Vivo

**FIGURE 1:**
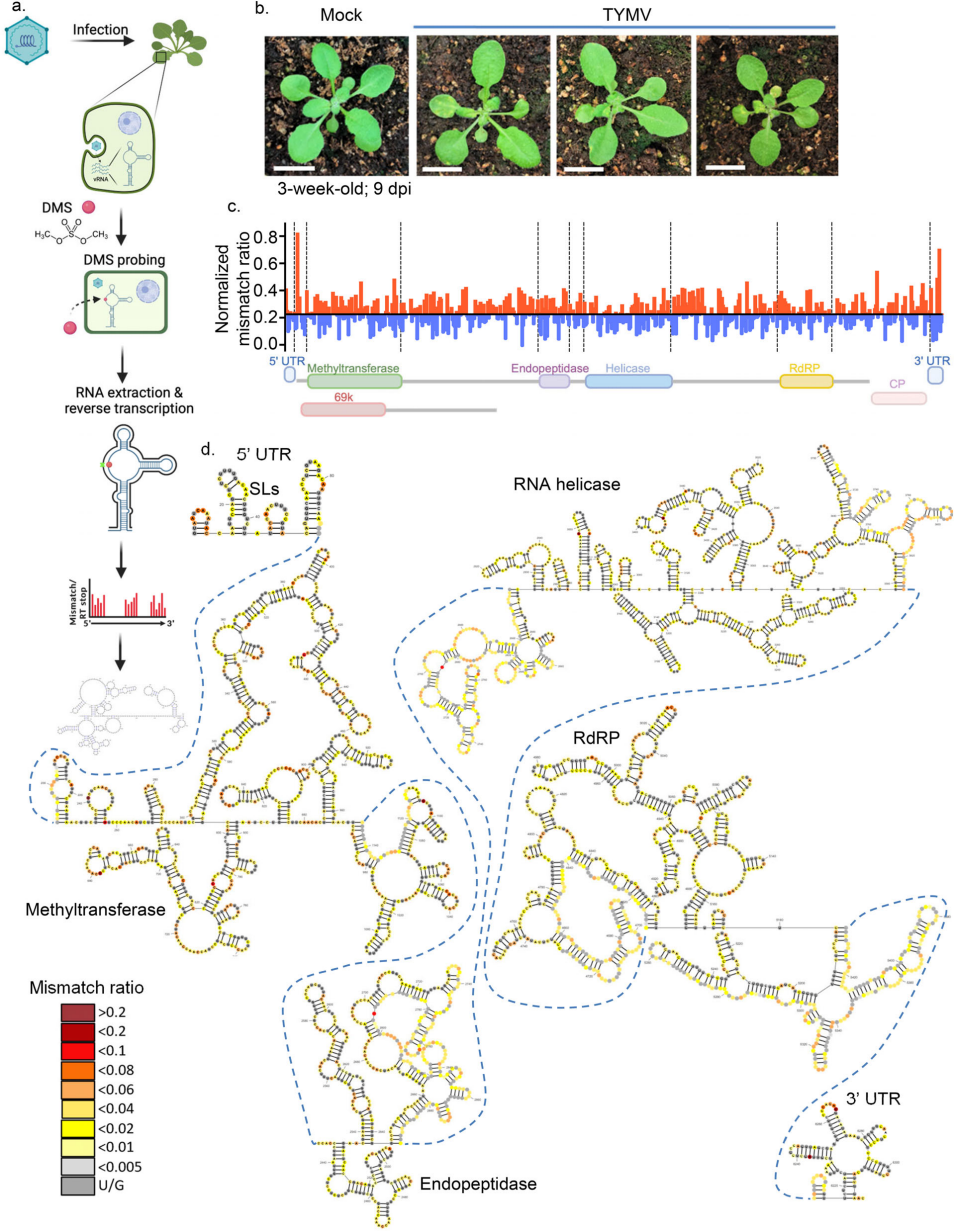
DMS‐MaPseq reveals the in vivo RNA structure landscape of Turnip Yellow Mosaic Virus. (a) Schematic of the experimental protocol for probing TYMV RNA structures in vivo using dimethyl sulfate mutational profiling with sequencing (DMS‐MaPseq). (b) TYMV infection results in yellowing and curly leaves. Photographs of TYMV‐inoculated Col‐0 at 9 dpi (days post‐inoculation). Scale bars, 1 cm. (c) Normalized mismatch ratio per nucleotide across the TYMV genome. The dashed line represents the domains that are translated from TYMV genome. In the top panel, the orange color represented the nucleotides above average mismatch ratio. The blue color showed the nucleotides below the average mismatch ratio. The data is calculated from merged replicates (n = 3). The bottom panel showed the TYMV genome organization, highlighting UTRs and protein coding regions. (d) Structural models of UTRs and protein domains of 206K, the longest ORF on the TYMV genome. The nucleotides are color‐coded based on DMS mismatch ratio. The regions without nucleotides labeling represent flanking regions of domains.

We investigated the TYMV RSS in vivo at a single‐nucleotide resolution by performing DMS‐MaPseq in TYMV‐infected Arabidopsis (Figure 1a,b). Briefly, we collected the entire infected plants 9 days post‐inoculation (dpi) when the yellow mosaic symptoms emerged in young leaves (Figure 1b). We treated the infected plants with DMS, an RNA structure‐probing reagent that selectively reacts with the single‐stranded adenine (A) and Cytosine (C) residues. The modified RNAs were extracted and subjected to reverse transcription, during which Thermostable Group II Intron Reverse Transcriptase (TGIRT), a reverse transcriptase that can introduce mismatches at modified nucleotides (Figure 1a), was used. We then performed high‐throughput sequencing of cDNA libraries and obtained sequencing reads of high quality, as the quality scores across all bases in all three replicates were all above 30 (Figure S1a). Furthermore, all sequence lengths of cDNA libraries uniformly reached the 150‐nt limitation in the paired‐end 150‐bp sequencing setting, indicating no significant trimming or premature read termination (Figure S1b). We then mapped the DMS mismatches across the viral genome and calculated DMS mismatch ratios at individual nucleotides, referring to DMS reactivity, which negatively correlates with the base‐pairing probability [8]. In the DMS‐MaPseq analysis, significant increases in mismatch ratios at A and C but not in G and U in all DMS‐treated samples confirmed the success of the DMS treatment (Figure S1c). We performed Pearson correlation analysis and found that inter‐replicate correlation values are higher than 0.99 for comparison of DMS reactivities of all A/C nucleotides (Figure S1d). All the above results further suggested that the DMS‐MaPseq is highly reproducible.

The average DMS reactivity varied markedly across the TYMV genome (Figure 1c). We used the RNAstructure program [34] to construct the in vivo structure model for the whole TYMV genome, using the bulk DMS mismatch ratio as a thermodynamic folding constraint. First, we modeled the U1‐snRNA, which has a well‐known conserved structure in human and rice [35, 36]. The modeled structure (Figure S1g) was highly consistent with previous studies in these two species (Figure S1e,f). To further validate the robustness and functional relevance of DMS‐MaPseq data in modeling the RSS in vivo, we calculated a Receiver Operating Characteristic (ROC) curve and corresponding Area Under Curve (AUC) between our U1‐snRNA DMS mismatch data and the published crystal structure of human U1‐snRNA. Such comparison yielded a favorable concordance, indicative of the strong rigor of the DMS‐Mapseq (AUC = 0.713) (Figure S1h).

Subsequently, we used the data to model the TYMV RSS (the coding region of the longest ORF in Figure 1d; overall structure of the whole genome in Figure S2a). The average mismatch ratio in single‐stranded and double‐stranded regions showed a significant difference (Figure S2b), indicating most of the nucleotides were assigned to single‐stranded/double‐stranded regions according to their mismatch ratio. Because the RNA structural modeling is more reliable for small RNA fragments (no longer than 4000 nucleotides (nt) as specified in the RNAstructure web server), we used a sliding window (window = 2000 nt, step = 100–200 nt depending on where one folding domain ends) to more accurately model RNA structures of a limited length. For overlapping regions, we selected structure models that share more similarities between the two fragments. To assess if the window size affected the modeling, we also used 700–900 nt windows with a step of 100–300 nt to model the RSS and compared it with the results of longer windows (Figure S3). Most of the RSS were not affected by the window size, despite the fact that the shorter‐window method tended to generate more end‐to‐end base pairings than the longer‐window algorithm.

Overall speaking, the TYMV genome contained independent RNA folding domains that expanded through the overall genomic backbone. These domains included numerous small stem loops and approximately 21 large and complicatedly folded structures across the genome, comparable to those of the HIV‐1 genome [14]. The average DMS reactivities in single‐stranded regions and double‐stranded regions showed a significant difference (Figure S2b). In the 5’ UTR (1–88 nt), we observed four short‐stem loops (labeled as SLs in Figure 1d; Figure S1). Following the cluster of four stem‐loop structures was another cluster of six stem loops that could extend to 292 nt in the genome.

The largest ORF of TYMV encodes methyltransferase, endopeptidase, RNA helicase, and RdRP in order (Figure 1d). The locus of methyltransferase, which spans from 207 to 1113 nt of the genome, formed four stem‐loop structures, one dumbbell structure, and three multiway junction structures. The endopeptidase coding region is 301‐nt long (2431–2732 nt of the genome). It had a four‐way junction, a longest stem‐loop structure, and a multiway junction structure. The last structure domain (multiway junction) covered the whole linker between endopeptidase and RNA helicase, and extended to the first 24 nt of RNA helicase. The RNA helicase region had four multiway junction regions and a cluster of six stem loops. The RdRP coding region formed three multiway junction structures and a 3‐bp stem‐loop. In an early study on HIV‐1 RSS, it was observed that the unstructured protein loops (including protein‐protein junctions and domain‐domain junctions) are derived from highly structured RNA elements. The observation, together with the reports that highly‐structured RNA slows and causes ribosomal pausing during translation, suggests that the highly‐structured RNA at protein‐domain junctions might stall ribosomes to allow time for protein or domain folding [14]. We predicted the structured and unstructured regions of both 206Kand 69K proteins using PrDOS [37], and aligned the DMS reactivities with the prediction results or domain‐linker regions. However, we did not see similar patterns in the TYMV genome (Figure S4a,b). In summary, we provided accurate DMS‐profiling data for the TYMV genome, which can serve as a starting point for identifying previously unrecognized functional elements.

### 2.2 RSS Heterogeneity is Prevalent Across the TYMV Genome

**FIGURE 2:**
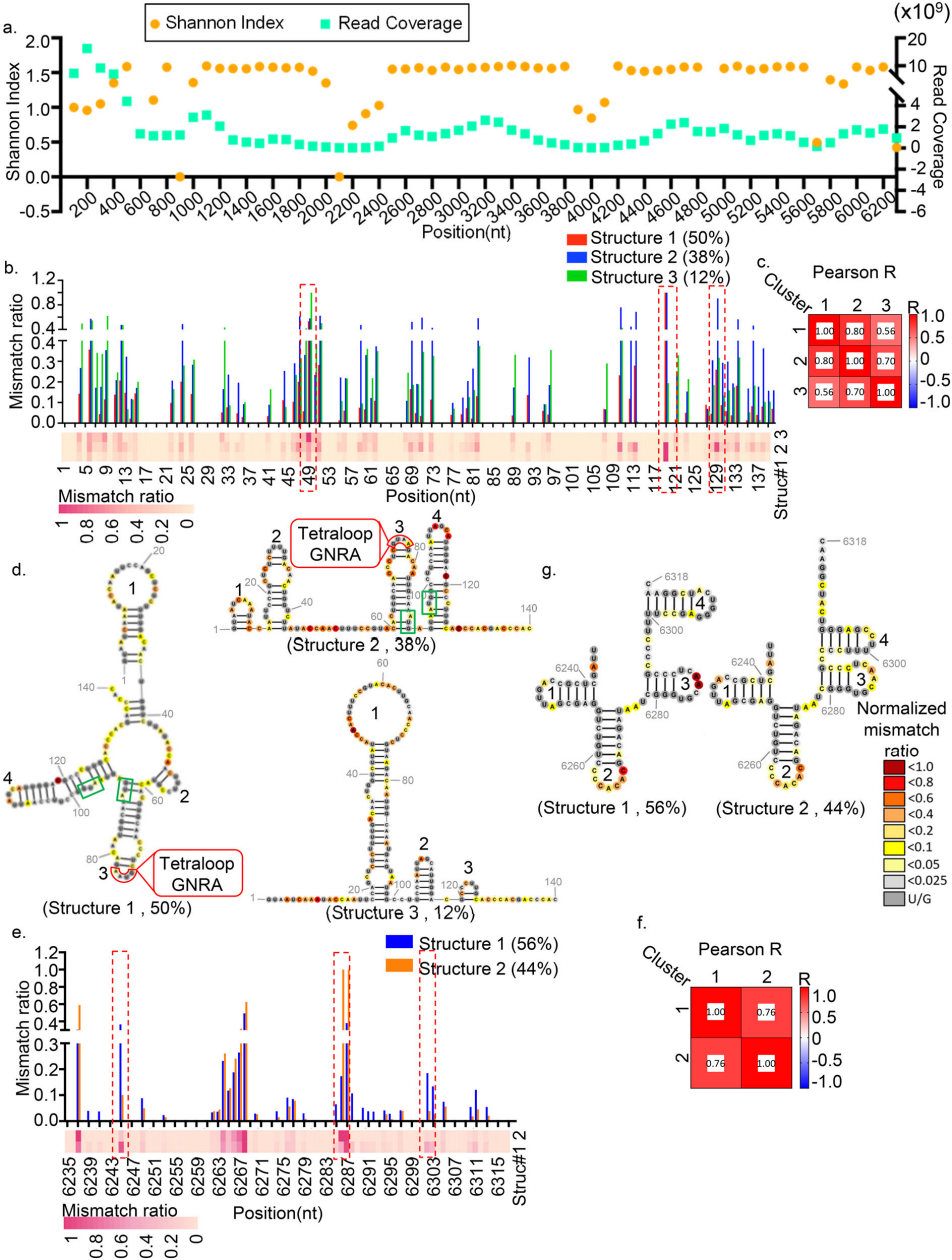
DREEM analysis shows the prevalence of RNA structural heterogeneity across the TYMV genome. (a) Landscape of RSS heterogeneity of TYMV. The Shannon index is shown as orange dots and the read coverage is shown as green dots. Each dot represents a 100‐nt window of DMS‐MaPseq data for DREEM algorithm. The data is from merged replicates (n = 3). (b) Normalized mismatch ratio for structures #1–3 of 5’ UTR. Only positions of A and C are shown. The heat map on the bottom compares the mismatch ratios of three structures in all positions. The positions with obvious differences among the three structures are labeled by red dashed frames. (c) Pearson correlation among three structures of 5’ UTR. (d) Models of three structures in 5’ UTR. The nucleotides are color‐coded according to normalized mismatch ratios. The numbers in gray represent the nucleotide positions on the TYMV genome. The green squares in Structure #1 and #2 represent the AUG of 69K and 206K. (e) Normalized mismatch ratio for structures #1 and #2 of 3’ UTR. Only positions of A and C are shown. The heat map on the bottom compares the mismatch ratios of two structures in all positions. The positions with obvious differences between two structures are labeled by red dashed frames. (f) Pearson correlation between two structures of 3’ UTR. (g) Structural models of two structures in 3’ UTR. The nucleotides are color‐coded as normalized mismatch ratio. The numbers represent the nucleotide positions on the TYMV genome.

It is believed that the RNA structure undergoes conformational changes in vivo, especially for multifunctional RNAs. Decoding RNA structural heterogeneity is very critical to fully understand the rules between RNA structures and their functions. Although we modeled RSS using the bulk DMS‐MaPseq data, we observed some paired regions with unexpectedly high DMS reactivity (some examples are labeled by pink arrows in Figure S2). We hypothesized that RNA structure in these regions might be heterogeneous. To test this, we adopted a clustering algorithm of “detection of RNA folding ensembles using expectation‐maximization” (DREEM) [20]. The principle for this algorithm is that if two individual DMS‐reactive bases are never detected on a single read, then the DMS reaction must take place in RNA with two different conformations. Thus, we divided the whole viral genome into 100‐nt with overlapping windows to determine whether any regions of the virus genome harbor alternative structures. We also introduced the Shannon index, which originally represents the weighted geometric mean of the proportional abundance of the types, to calibrate the RNA structure diversity in our analysis [20]. A Shannon index of 0 indicates homogeneity whereas a higher value refers to an increased heterogeneity. In this scenario, we observed that a few loci exemplified by nucleotides 800–900 and 2000–2100 displayed only one form, suggesting the homogenous structures in the regions (Figure 2a). However, more than 96% of windows had Shannon indices of approximately 1.5, indicative of the presence of more than one cluster of RSS in the regions (Figure 2a). Thus, the DREEM analysis shows the wide prevalence of heterogeneity in RNA structure across the whole TYMV genome. In lines with this observation, the RSS of the majority of HIV genome also forms more than one alternative structure [20], suggesting that RSS heterogeneity could be a common feature of viral RNAs.

We then focused on the 5’ UTR and 3’ TLS of TYMV, which are believed to possess certain regulatory functions. We found that the 5’ UTR displayed three alternative structures, named as Structure #1, 2, and 3, which shared aliquots of 50%, 38%, and 12%, respectively (Figure 2b). We further profiled the mismatch ratios at each nucleotide of the three alternative structures (Figure 2b). Clearly, the heatmap comparison indicated that mismatch ratios of some positions are remarkably different, such as position 49, 119, and 129 (Figure 2b, as shown in red dashed frames). We calculated the Pearson correlation among the three alternative structures, R value between structure #1 and #2 is 0.8, indicating their structure might be similar, while structure #3 has a lower R value than both #1 and #2 (Figure 2c). Structure #1 forms a compact structure, indicated by the lowest DMS signal among the three structures (Figure 2d). The very 5’ end of the structure contains a 32‐nt stem loop. It has been reported that insertion of a stem loop in the 5’ termini would abolish the translation of 206K and 69K [38]. Thus, we could postulate that structure #1 represents a non‐translational form. Besides, it has been noted that RNA of Dengue virus (DENV) is in a more circular form in the viral particles compared with the free RNA [39]. In addition, the RNA with a more compact RSS has a higher affinity to the viral coating proteins [40]. Analogous to this pattern, we speculated that the compact feature of structure #1 was in the viral capsid, which might further hinder the viral RNA from reacting with DMS (Figure 2d, structure #1). Structure #2 displayed the same hairpins 3 and 4 as structure #1. Interestingly, the start codons of 69K and 206K are hidden on hairpins 3 and 4, respectively (Figure 2d, green squares in structure #1 & #2). As reported previously that knockout of AUG of 69Kwould increase 206K expression and vice versa, the hairpin 3, which masks AUG of 69K, contained a canonical tetraloop structure GNRA. Such a configuration is assumed to make the hairpin 3 particularly stable as reported before [41]. While hairpin 4 does not have stabilizing elements. This kind of structure could allow the translation to start in the AUG of 206K with the priority to produce polyproteins essential for viral propagation in vivo because the hairpin 4 is more unstable than the hairpin 3. As reported previously, knockout of AUG of 69K would increase 206K expression and vice versa, indicating that the AUG of 69K and 206K are competitive [42]. Based on the structural configuration of hairpins 3 and 4, the AUG selection may depend on both RNA structure and sequence context. Notably, structure #2 is highly consistent with the previously reported 5’ UTR structure that was probed by RNase S1, RNase T1, and CMCT in vitro [43]. The consistency in vivo and in vitro indicated the structure is thermodynamically favorable. Instead of showing several hairpin structures, structure #3 contains a large stem loop and two short hairpins. As the hairpin stability decreases as the loop size increases, the large loop in structure #3 is expected to be unstable. The AUGs of 69K and polyproteins are both in the large stem‐loop. This organization would enable the 69K to be produced once the large stem‐loop is open.

The 3’ TLS has two alternative structures, which made up of 56% (structure #1) and 44% (structure #2), accordingly (Figure 2e,g). The Pearson correlation between two alternative structures is 0.76 (Figure 2f), indicating that these two structures are marginally different. The two structures are remarkably different from the RNA fold based on the bulk DMS‐MaPseq data (Figure S2a). Specifically, the structure modeled from bulk data did not show the typical tRNA‐like structure in the 3’ UTR as reported from in vitro X‐ray crystal or cryo‐EM approaches [26, 44]. By contrast, structure #1 identified by DREEM was consistent with the 2D structure of 3’ TLS characterized by X‐ray crystallography [26] (Figure S5). Importantly, we discovered another novel structure in the 3’ TLS, in which the pseudoknot is re‐organized to a stem‐loop. The profile patterns of the mismatch ratios at individual nucleotides of the two structures are clearly different (Figure 2e). Furthermore, the heatmap comparison of mismatch ratios pinpointed some positions with obvious differences in the DMS reactivity, such as position 6245, 6286, and 6302 (as shown in red dashed frames). We noticed two nucleotides (positions 6286 and 6287) that have unexpectedly high mismatch ratios in structure #1 of 3’ TLS. It was reported that the TGIRT notably produces mismatches at endogenous m^1^A and m^3^C tRNA residues, which are the methylated sites of a DMS modification [8]. We examined the control sample, which was not treated by DMS, and found that the mismatch ratios of these two nucleotides of the control sample are similar to the overall average mismatch ratio, which is not as high as the DMS‐treated samples. The results suggested that the high mismatches of the two nucleotides are not caused by m^1^A modifications. Taken together, we observed that the structures in 5’ UTR and 3’ TLS, which were previously identified in vitro, indeed existed in vivo. However, new alternative structures in 5’ UTR and 3’ TLS could be also identified in vivo via DMS‐MaPseq and DREEM analyses.

### 2.3 The Structure Conservation Through the Different Species of Viruses

**FIGURE 3:**
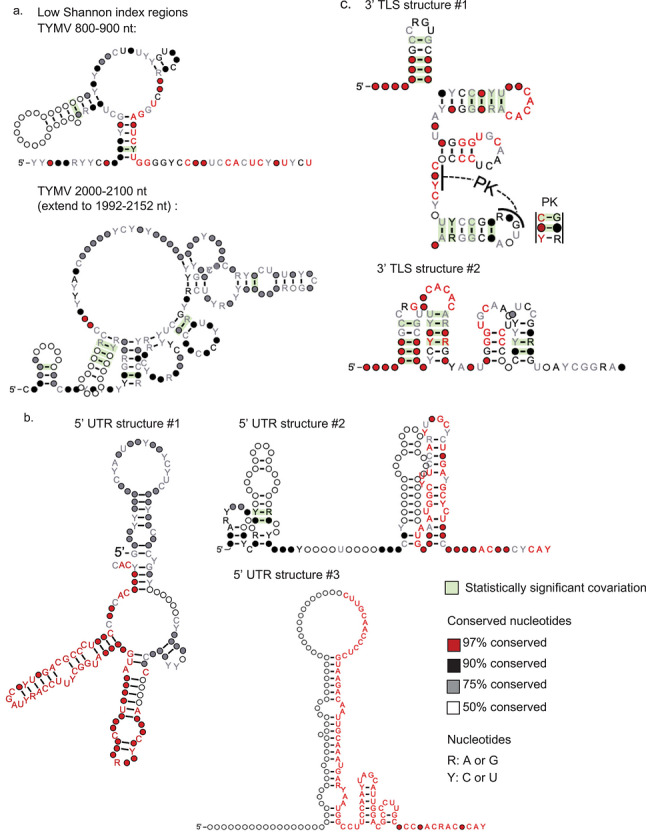
Conservation and covariance analysis reveal structural conservation on 3’ TLS. (a) Conservation and covariance analysis show that the lowest‐diversity regions contain numerous covariance pairs. The 2000–2100 nt region was extended to 1992–2152 nt to maintain the whole structural domain. (b) Conservation and covariance analysis of three different structures of 5’ UTR shows relatively less structural covariance. (c) Conservation and covariance analysis of two different structures of 3’ TLS shows high structural covariance. PK indicates pseudoknot.

The co‐evolution of an RNA structure suggests its functional conservation. We next examined the possible co‐evolutionary evidence in the 5’ UTR, 3’ TLS and two regions with the lowest RSS heterogenicity (800–900 nt and 2000–2100 nt as shown in Figure 2a). Briefly, we used Infernal package [45] to build a structure covariation model and searched for an alignment homologous sequences in *Tymoviridae* family, which includes TYMV, Eggplant Mosaic virus, and Andean potato latent virus among others. In the low‐diversity region of 800–900 nt, which forms a dumbbell structure, we found two covariance pairs among 16 base pairings, as shown in Figure 3a (top). In the low‐diversity region of 2000–2100 nt, we extended the region to 1992–2152 nt to include the whole structural domain. The RSS in this region is special because it forms a six‐way junction—the most branched junction across the genome. We found seven covariance base pairs in the region, which indicated that these base pairings could be conserved (Figure 3a, bottom). However, most of the base pairings within the structure are not covariable, suggesting that the conservation of the overall structure requires further evidence.

We observed low conservation in the three alternative structures of 5’ UTR, suggesting the RSS in 5’ UTR might change or still be under selection during evolution (Figure 3b). This might also imply that RNA sequence is more important for 5’ UTR function. By contrast, we observed a significant number of conserved base pairs in the two alternative structures of 3’ TLS (Figure 3c). In structure #1, 14 among 25 base pairs, including two base pairs in the pseudoknot, are conserved. We also observed 10 among 20 base pairs are conserved in structure #2. To be noted, Infernal's alignment and consensus sequences are heavily affected by the secondary structure constraints provided in the model. In the covariance analysis here, the same sequence with different structure annotations was used as input to construct covariance models (CM), which would weight sequence positions differently based on the structural context. This may lead to the different consensus sequence in each proposed conserved structure. Overall, the fact that the two alternative structures showed significant co‐evolutionary evidence strongly suggested that both structures are critical to viral pathogenicity.

### 2.4 The Alternative Structures in 3’ TLS are Indispensable for Viral Pathogenicity

**FIGURE 4:**
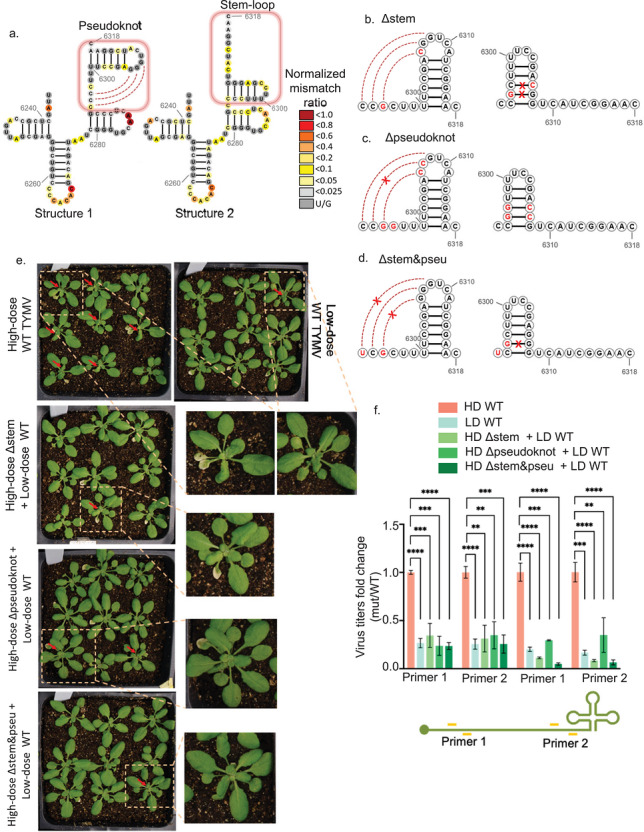
Alternative structures in 3’ TLS are indispensable for viral pathogenicity. (a) Structure models of 3’ TLS from DREEM analysis. The dashed line on Structure #1 represents the pseudoknot revealed by the reported in vitro X‐ray crystallography and NMR [26, 33]. The pink squares highlight the region of mutations in (c). (b) Design of TYMV mutant (Δstem) that aims to disrupt Structure #2. (c) Design of TYMV mutant Δpseudoknot that aims to disrupt Structure #1. (d) Design of TYMV mutant Δstem&pseu that aims to disrupt both Structure #1 and Structure #2. The nucleotide numbers on (b–d) correspond to the positions on (a). The cross on the hydrogen bond represents the disruption of the base pairing. The nucleotides in red represent the mutations. (e) Pathogenicity phenotypes of TYMV and its Δstem, Δpseudoknot and Δstem&pseu variants with a spike of 10% WT TYMV. The squares framed in dashed pink lines highlight the typical symptomatic plants and are also zoomed‐in pictures. Photographs were taken of the plants at 9 dpi (days post‐inoculation). Scale bars, 1 cm. (f) qRT‐PCR assays show the relative titers of WT or mutant viral RNAs from different inoculations at 9 dpi. The relative amount of viral RNA was first normalized to *UBC10* control, and then to that of HD WT TYMV where the mean was arbitrarily assigned a value of 1. The TYMV primers' relative locations are indicated at the bottom. HD represents high‐dose TYMV/mutants; LD represents low‐dose TYMV/mutants.

To experimentally investigate whether two alternative RNA structures in 3’ TLS were critical to viral pathogenicity, we designed several mutations to perturb the population of RNA structures (Figure 4b–d). The relative position (nucleotide 6295–6318) in Figure 4b–d was shown in the red square in Figure 4a. First, we swapped a G‐C base pair in the pseudoknot region of 3’ TLS (Δstem mutation) and such mutations were expected to disrupt structure #2 but maintain structure #1 (Figure 4b). Then, to maintain structure #2 but disrupt structure #1, we swapped two G‐C base pairs in the stem region of structure #2, and such mutations were assumed to disrupt the pseudoknot in structure #1 (Δpseudoknot mutation, Figure 4c). Finally, to completely disrupt both alternative structures, we mutated the first C and last C in the pseudoknot region to U and G, respectively, and these changes were expected to disrupt both structure #1 and structure #2 (Δstem&pseudoknot, Figure 4d). To validate the structural mutations, we initially planned to perform DMS‐MaPseq for the three virus mutants. However, the Δstem, Δpseudoknot, and Δstem&pseu mutants failed to infect the plants, leading to extremely low virus titers and precluding the effort of in vivo RSS profiling of the variants. Instead, we carried out in vitro DMS‐MaPseq of in vitro transcribed and folded transcripts of WT and the three viral mutants. Indeed, the Δstem mutation disrupted the stem‐loop structure and adopted a consistent conformation with the pseudoknot structure (Figure S6a). On the other hand, the Δpseudoknot mutation disrupted the pseudoknot structure and partially adopted a stem‐loop‐like structure, forming two stem‐loops within the final 45 nucleotides (Figure S6b). Although the Δpseudoknot was not an exact replica of the stem‐loop structure, the disruption of the pseudoknot and emergence of stem‐loop‐like conformation indicated that the mutations successfully shifted the folding equilibrium toward the intended conformation. Finally, the Δstem&pseudoknot mutant disrupted both alternative structures, consistent with the design intention (Figure S6c). Taken together, the DMS‐MaPseq in vitro largely validated the mutagenesis designs and would provide meaningful structural insight into the virus mutants.

To assess whether the mutations specifically affected the 3’ TLS region without altering the RSS of other regions, we calculated the Pearson correlation coefficients for the DMS mismatches among WT, Δstem, Δpseudoknot and Δstem&pseu in the three regions of the virus: 1) 3’ TLS (6235–6318 nt); 2) an upstream control region immediately preceding the 3’ TLS (6150–6234 nt) and, 3) the remainder of the virus genome excluding the 3’ TLS (1–6234 nt). The Pearson correlations in both control regions were consistently high (R>0.9, Figure S6e,f), indicating that these regions were largely unaffected by the mutants. In contrast, the Pearson correlations in the 3’ TLS were substantially lower (Figure S6d), reflecting significant structural perturbations induced by the mutations. These findings suggested that the structural mutations specifically disrupted the RSS within 3’ TLS, while leaving upstream and genome‐wide RNA structure largely unchanged.

The above‐mentioned 3’ TLSs with different structures were used for further functional assays. Briefly, we in vitro transcribed the full‐length of wild‐type (WT) viral RNA and variants with different 3’ TLSs and infected the plants with identical dosages of the transcripts. Then, we examined the infectious capacity of WT and the mutant viruses by counting the number of symptomatic plants and measuring the virus titers via qRT‐PCR. Interestingly, the WT viral RNAs were able to infect the Arabidopsis readily, while the three mutants lost their infection capacity (Figure S6g). To further pinpoint the steps where the mutant viruses lost their pathogenicity, we spiked 10% of WT viral RNA into the mutant viral RNA to initiate the production of necessary proteins for viral propagation. In this scenario, we observed a high infection rate of WT virus, while the mutant viruses showed similar infection rates to that of the low‐dose WT virus (Figure 4e; Figure S6h). In lines with the phenotypic difference, qRT‐PCR assays showed that all mutants had dramatically decreased viral titers compared with WT virus (Figure 4f), indicating that both structures in the 3’ TLS were essential for virus infectivity. Taken together, we concluded that the alternative structures in 3’ TLS structure of TYMV were indispensable for viral pathogenicity.

### 2.5 The Pseudoknot Structure in 3’ TLS Facilitates Viral Translation

**FIGURE 5:**
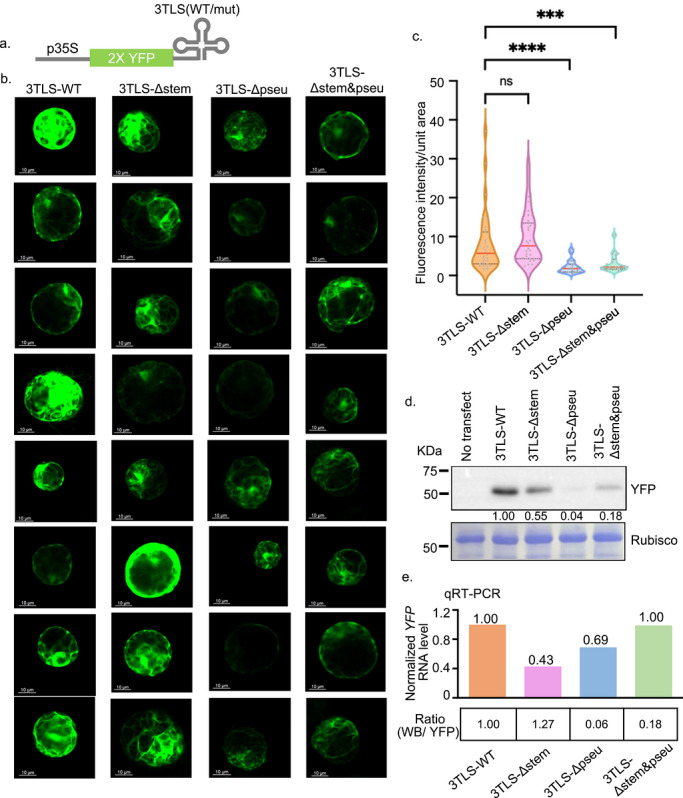
The pseudoknot structure in 3’ TLS facilitates viral translation. (a) Schematic of the *2xYFP‐3’ TLS* reporter. (b) Representative YFP signal of protoplasts transfected with the *YFP‐3’ TLS‐WT*, *‐3’TLS‐Δstem*, *‐3’ TLS‐Δpseudoknot* and *‐3’ TLS‐Δstem&pseu* constructs. (c) Statistical analysis of *YFP* signal intensity of individual protoplasts. Each dot in the plot represents one protoplast. The red horizontal line represents the median. Gray horizontal lines represent 25th and 75th percentiles (*n*  =  30 biologically independent protoplasts). P values were calculated by one‐way ANOVA analysis with Tukey's multiple comparisons test. (d) Western blot analysis shows protein levels in the protoplasts. The signals were quantified by image J where WT was arbitrarily assigned a value of 1. Coomassie blue staining of Rubisco serves as a loading control. (e) qRT‐PCR assay shows RNA levels of *YFP* in the protoplasts. The *YFP* and *Hygromycin* RNA levels were first normalized to the internal control *UBC10*, and then the *YFP* RNA level was normalized to the *Hygromycin* RNA level, which was co‐expressed in the same plasmid with *YFP*. The *YFP*/*Hyg* ratio of the reporter fused with WT 3’ TLS was arbitrarily assigned a value of 1. The protein levels detected from the western blot were then normalized to the *YFP* RNA levels, and the ratios were labeled at the bottom. The protoplast transfection experiments were independently repeated three times, with consistent results. The replicates were shown in Figure S7a–d.

It has been reported that the aminoacylation on 3’ TLS is critical in replication [27, 28, 29, 30] and translation [31, 32]. However, it is unclear whether the alternative structures in 3’ TLS contribute to channeling the viral RNA into different biological processes. We hypothesized that one structure on 3’ TLS facilitates translation, whereas the other one might promote replication. To test the hypothesis, first we constructed a reporter, fusing the *YFP* with WT or mutated viral 3’ TLS to examine whether structural changes on 3’ TLS affected translation (Figure 5a). The constructs were then delivered to protoplasts. We randomly selected 30 protoplasts to observe the YFP signal under the confocal microscope. Intriguingly, a significantly reduced YFP signal was observed in the reporters with Δpseudoknot and Δstem&pseu, where the pseudoknot structure was disrupted (Figure 5b,c). However, the reporter with only Δstem did not show a decrease in the YFP signal. Since the mutations on Δstem only swapped the C‐G to G‐C pairing and should maintain the base pairing of the pseudoknot structure, the results suggested that the pseudoknot structure was critical for protein synthesis. To validate this, we extracted the proteins and RNAs from the protoplasts. *YFP* protein level was quantified by western blot analysis (WB). The *YFP* RNA level was quantified by qRT‐PCR, with *UBC10* serving as internal control, and normalized to the amount of *Hygromycin* RNA, which was co‐expressed from the same construct. The WB signal was then normalized to the *YFP* RNA level to exclude potential variations among replicates. Indeed, the WB signal to *YFP* ratio were decreased in the Δpseudoknot and Δstem&pseu mutants, while the Δstem mutant did not show a significant change (Figure 5d–e; Figure S7a–d). The result was consistent with the YFP signal under the confocal microscope, suggestive of the essential role of pseudoknot structure in translation.

### 2.6 The Structural Dynamics in 3’ TLS Modulate the Viral Replication

**FIGURE 6:**
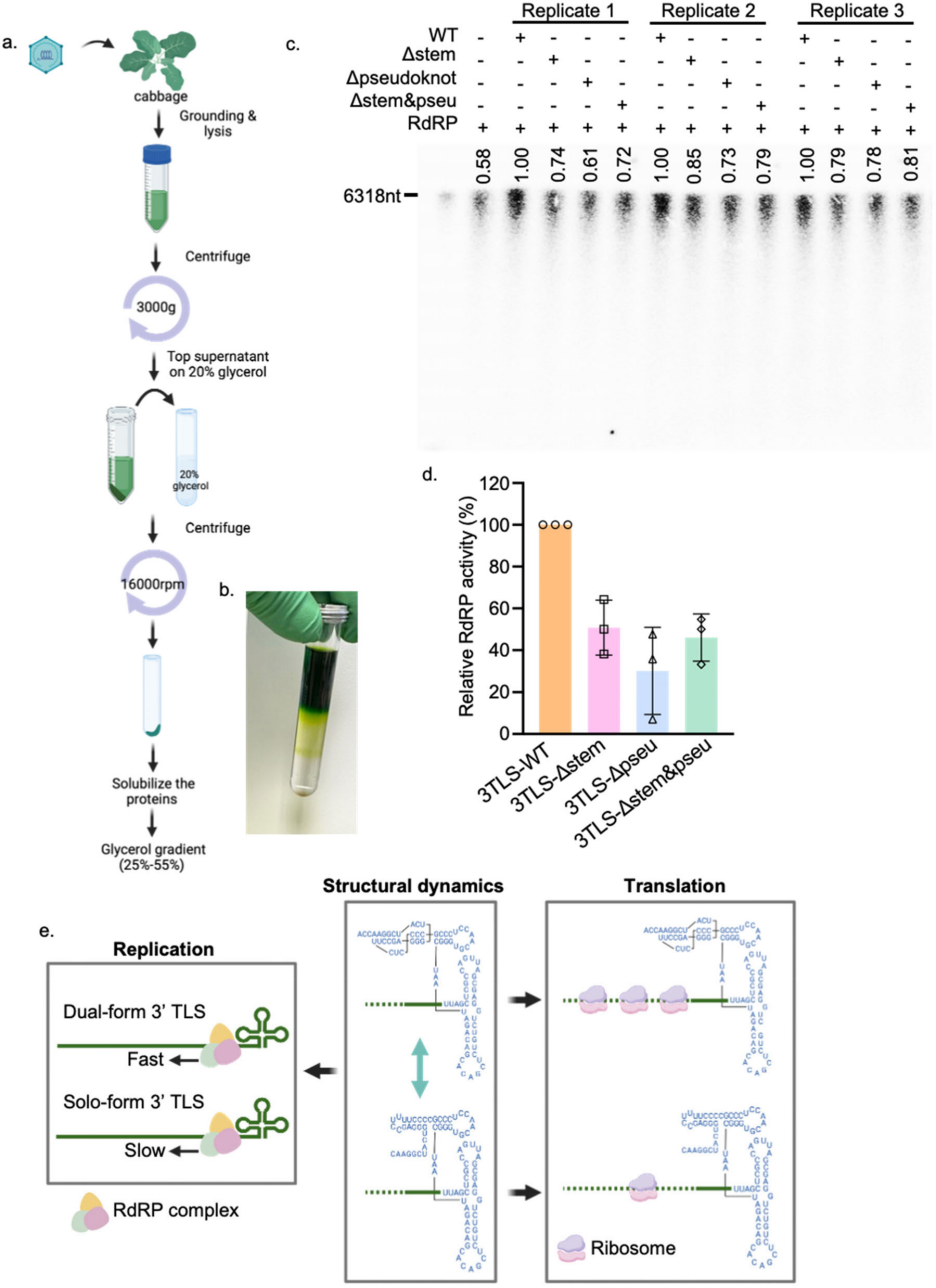
The structural dynamics in 3’ TLS is essential for the viral replication. (a) Experimental schematic of RdRP complex purification from inoculated cabbage. (b) Photograph after glycerol gradient ultracentrifuge in (a). (c,d) RdRP assays without or with different RNA templates. The reaction took place with the presence of [α‐^32^P]UTP. The products of three independent replicates were run on the same urea‐PAGE gel and shared the same negative control. The signals were detected by phosphor imaging and then quantified by image J where WT was arbitrarily assigned a value of 1. Quantification of relative RdRP activity as mean ± SD from three replicates was shown in (d). Be noted: in the negative control, some basal activity of RdRP could be detected due to the residual RNA present in the complex, a common pattern when crude RdRP complex is reconstituted from the virus‐infected materials [46]. (e) A proposed model for RSS function of 3’ TLS in replication and translation. The RSS of 3’ TLS is dynamics in vivo. The pseudoknot structure facilitates translation, while the loss of RSS heterogeneity compromised viral replication.

The results above (Figure 5) could not fully explain why all the mutants in 3’ TLS lost the infection capability. We wondered whether the structural mutations in 3’ TLS would affect the viral replication. To test this, we performed in vitro virus replication reconstitution assays. First, we purified the RdRP complex from the infected plants by glycerol gradient ultracentrifugation (Figure 6a,b). Since RdRP often displays dual activities: RNA‐dependent RNA polymerase and terminal nucleotide transferase (TuTase), we first optimized the reaction conditions for monitoring virus replication. To this end, we collected four fractions from the glycerol gradient ultracentrifugation for testing RNA‐dependent polymerase and TuTase activities in parallel (Figure S8a). Briefly, each fraction was added to two reaction conditions while containing the same RNA template: one contained all four NTP (including [α‐^32^P]UTP), and the other had [α‐^32^P]UTP only. The reation products were fractionated in a urea‐PAGE gel after being vigorously washed with phenol/chloroform. The results showed that fraction #1 yielded a significantly stronger signal compared to the other fractions. Furthermore, the reaction with [α‐^32^P]UTP only produced a marginally detectable signal compared to the one with the full set of NTPs (Figure S8b). These results indicated that the observed products from the fraction #1 are primarily from RdRP activity rather than the TuTase activity.

Based on the results, full‐length WT or mutant virus transcripts were folded in vitro and then incubated with the purified RdRP complex (the fraction #1) in the presence of [α‐^32^P]UTP. Because the RdRP complex binds viral RNA tightly in vivo, even though we provided micrococcal nuclease during RdRP purification to remove potential single‐ and double‐stranded RNA and DNA contaminations, we still observed a background signal from endogenous viral RNAs in the reaction without providing RNA templates. The presence of residual background signal is common when crude RdRP complex is reconstituted from virus‐infected materials [46]. Importantly, when we added WT viral templates, we observed a significant increase in α‐^32^P signal in products, indicating that the RdRP complex could replicate transcripts efficiently using WT viral RNA as templates. However, when the Δstem, Δpseudoknot and Δstem&pseu viral RNAs were provided as templates, the signal of newly synthesized transcripts significantly decreased, no matter which structures were disrupted (Figure 6c,d). Our results indicated that both structures were critical to viral replication, with a suggestion that the RNA structure dynamics is essential for the virus replication. Otherwise, perturbing the balance between alternative structures would significantly influence viral replication.

## 3 Discussion

In this study, we profiled the RSS of TYMV and analyzed the RSS diversity across the TYMV genome in vivo. To our best knowledge, it is the first in vivo genome‐wide RSS for a plant virus. The structural models revealed the complexity of RSS across the virus genome, most of which were unknown previously. These complicated structural features of RNA genome might harbor various regulatory layers or hubs to control virus replication and/or protein synthesis. In fact, emerging evidence has shown that the RSS of a few mammal viruses, such as HIV, influenza, dengue, ZIKV, and coronavirus hold the key for the regulation of viral infection. For example, the discovery of a pioneer study of the HIV RNA genome structure suggested the higher‐order RNA structure directly encodes protein structures [14]. Other studies found xrRNA pseudoknot structures in 3’ UTR of Dengue, ZIKV, and other flaviviruses stall the RNA exonuclease Xrn1 to facilitate the production of non‐coding sub‐genomic flavivirus RNAs [47, 48].

In our RSS model of TYMV, we found the protein‐coding regions harbor extensive structures. Although the viral dsRNA can be recognized by *Dicer‐like* (*DCL*) proteins and cleaved into small interfering RNAs (siRNAs) to silence the viral RNAs, the extensive RSS on the genome also provides a platform for the RNA binding proteins to regulate the virus pathogenicity. In addition, as the RSS should be unwound during translation and replication, RNA helicases, yet unidentified, are likely involved in regulating these processes. Since it is very challenging to dissect the functional significance of RSS in the protein‐coding loci, we selected the UTRs as examples to conduct in‐depth analysis for the importance of RSSs. We found alternative RSS is pervasive, including the 5’ UTR and 3’ TLS regions of TYMV. Different from the prevailing view that the single homogenous structure in 3’ TLS involved in viral translation and replication, we found that alternative structures that naturally exist in vivo may participate in different biological processes. It has been revealed that alternative RNA conformations determine whether a transcript is spliced [20] and whether the RNA bind or not bind to proteins [44, 49]. In this study, we found that alternative structures function in viral translation and replication. To be specific, the alternative RSS (pseudoknot form) in 3’ TLS of TYMV functions in translation but not in replication, while both the pseudoknot form and the stem‐loop form (newly‐found structure in this study) of 3’ TLS function in replication. The result highlighted the importance of the RNA structural dynamics and equilibrium, even in a single biological process. It also implies that different structures may channel the RNA into different stages such as initiation and elongation during replication. The higher replication efficiency exhibited by the dual‐structure dynamics relative to either solo structure suggests strong functional complementation between the two 3’ TLS structures during viral RNA replication. Viral replication consists of at least two distinct steps: initiation, during which the RdRP complex is efficiently recruited to the 3’ terminus, and elongation, during which RdRP proceeds along the template to synthesize the negative strand. The co‐existence of two different structures in vivo would enable dynamic and swift conformational switching between the two functionally distinct states. In contrast, either solo conformation might be functionally incomplete and insufficient for maximal replication.

Besides translation and replication, the 3’ TLS structural dynamics may also modulate viral infection through additional regulatory layers. Since the RNA structure is known to affect the DCL accessibility and recognition [1], and viral siRNAs are distributed throughout the TYMV genome, including the 3’ TLS region [50], we cannot exclude the possibility that the 3’ TLS conformation may also affect host activity in posttranscriptional gene silencing. In addition, the pseudoknot‐containing conformation promotes efficient translation of viral proteins, including P69, a critical viral suppressor of RNA silencing. These potential regulatory mechanisms may act coordinately with translation and replication to determine overall infection efficiency, and deserve further investigation in the future.

The multilayered regulatory events illustrate how viral RNA structures fine‐tune diverse steps of infection to maximize viral pathogenicity. The efficient propagation of most RNA viruses is determined by how efficiently they exploit the host machinery to finish the initial life cycle because viruses require a host cell to make new products. Sophisticated RNA structures serve as a large platform for regulation, which may involve in fundamental processes such as subcellular location, translation, stability, replication, and et al. Since more and more functional virus RSS motifs are revealed, innovative antiviral strategies will be developed based on RSS. Our data provide a robust and comprehensive map for potential antiviral drug designs to combat the invaders. Especially the structure in tRNA mimic region which has been reported in human viruses [21, 51] such as HIV‐1, would provide valuable insight to developing an effective anti‐viral strategy.

## 4 Method and Materials

### 4.1 Plant Materials Growth Conditions

In this study, *Arabidopsis thaliana* (*A. thaliana*) Col‐0 ecotype was used in all virus infection experiments. *A. thaliana* plants were grown under 22°C with short‐day condition (8 h light/16 h dark) unless addressed specifically.

### 4.2 Plasmid Constructs

For full‐length TYMV, we separately cloned three fragments from the cDNA of TYMV‐infected plants into pENTR vector using the primers listed in the Table S1. The first forward primer contained a T7 promoter, and the last reverse primer contained a HindIII restriction site. After sequence verification of the positive clones, we assembled the full‐length viral sequence from the three pENTR clones using the intrinsic restriction endonuclease sites on the viral genome. The Δstem, Δpseudoknot and Δstem&pseu mutant forms were obtained by site‐directed mutagenesis by PCR using fully overlapping forward and reverse primers which have substitutions of the specified nucleotides. The resulting vectors were all confirmed by sequencing.

*P_35S‐_2XYFP‐3’ TLS* was constructed as follows: The *YFP* and 3’ TLS, including wild‐type (WT) 3’ TLS and mutant 3’ TLS, were amplified separately using the primers listed in Table S1 and different templates harboring the mutation. The PCR fragments were cloned into pENTR vector using NEB HiFi DNA Assembly Master Mix followed by sequencing confirmation. Then by Gateway attL‐attR (LR) reaction, *YFP‐3’ TLS* was transferred into a binary vector *P_35S_‐YFP‐DC* to obtain *P_35S_‐2XYFP‐3’ TLS*. The primers for all constructs are listed in Table S1.

### 4.3 TYMV Inoculation Assays

For DMS‐MaPseq, 12‐day‐old plants were inoculated by inoculum (diseased plants) in 10 µ Potassium Phosphate buffer (pH 7.0) by gently brushing one of the first pair of true leaves. The materials were collected at 9 day‐post‐inoculation (dpi). For the pathogenicity examination experiment of WT, Δstem, Δpseudoknot, and Δstem&pseu mutants, the same amount of in vitro transcribed full‐length viral RNAs in inoculation buffer (30 mm K_2_HPO_4_, 50 mm Glycine, 5 g/L Bentonite, 5 g/L Celite) were rubbed on one of the first pair of true leaves. The symptoms were recorded, and materials were collected at 7 dpi.

### 4.4 In Vivo DMS Treatment

In vivo DMS treatment of the plants was performed as described with modifications [6]. Briefly, the samples were harvested and immersed in 25 mL DMS reaction solution (40 mm HEPES pH7.5, 100 m KCl, and 0.5 m MgCl_2_). 1% DMS (v/v) was added. For negative control, the same volume of DMSO instead of DMS was added. The samples were treated under vacuum at room temperature for 15 min, with swirling every 7.5 min. The reaction was quenched with the addition of β‐mercaptoethanol (final concentration 20%, v/v) and vacuum for 5 min. Wash the samples three times with 50 mL distilled water. Freeze the samples with liquid N_2_ and grind to fine powder.

### 4.5 RNA Extraction

The plant materials were grinded into fine powder. 0.1 g powder was mixed with 1 mL of TRI reagent (Sigma–Aldrich, cat#T9424) and total RNA was extracted per the manufacturer's manual. Denatured MOPS agarose gel electrophoresis was used to validate the integrity of RNA.

### 4.6 In Vitro Transcription of Biotinylated Anti‐Chloroplast RNA Probes

To increase the sequencing depth of targeted transcripts, we made several probes to remove the highly expressed chloroplast genes. The probes were designed as described previously with modifications [35]. Targeted chloroplast genes were amplified using primers containing T7 promoter listed in Table S1 and cloned into pENTR vector. The resulting plasmid constructs were sequenced and employed as the PCR templates to amplify sequences for in vitro transcription. The PCR products were gel purified using Qiagen QG (cat#19063) and PE (cat#19065) buffer. The in vitro transcription reaction containing 250 ng templates, 1 µL of 100 mm ATG, 1 µL of 100 m CTP, 1 µL of 100 m GTP, 0.4 µL of 100 m UTP, 0.8 µL of 50 m biotin‐16‐UTP (Lucigen), 1.5 µL T7 RNA polymerase (lab purified), and 1 µL Superase‐In RNase inhibitor (Invitrogen cat#AM2696) in a total of 20 µL was incubated at 37°C overnight. Then, 1 µL Turbo DNase (Invitrogen cat#AM2239) was added and incubated at 37°C for 30 min. Finally, RNA probes were purified by RNAClean XP beads (Beckman #A63987). The RNA probes were combined with a final working concentration of 160 ng/µL anti‐*PSBA*, 70 ng/µL anti‐*RBCL*, 15 ng/µL anti‐*PSAB*, 15 ng/µL anti‐*PSAA*, 10 ng/µL anti‐*PSBC*, 10 ng/µL anti‐*PSBB*, 10 ng/µL anti‐*PSBD* probes. These anti‐chloroplast probes were mixed with rRNA removal mix (Illumina Truseq Stranded Total RNA with Ribo‐Zero Plant kit) to remove contamination of highly expressed chloroplast RNA and rRNA.

### 4.7 rRNA and Chloroplast RNA Depletion

The process was modified from the manual of Illumina Truseq Stranded Total RNA with Ribo‐Zero Plant kit and established protocol previously [35]. Total RNA was extracted by TRI reagent from TYMV‐infected or mock plants after treatment of 1% DMS. RNA was subsequently subjected to Turbo DNase treatment for 1 h at 37°C. rRNA was depleted by Illumina Truseq Stranded Total RNA with Ribo‐Zero Plant kit and chloroplast RNA was depleted by in vitro transcribed RNA probes. Briefly, 1 µg DNase‐treated total RNA was mixed with 1 µL anti‐chloroplast RNA probes mixture, 5 µL rRNA binding buffer (Ribo‐Zero Plant kit), and 5 µL rRNA removal mix (Ribo‐Zero plant kit) in a total of 20 µL. The mixture was heated at 65°C for 5 min and then gradually decreased the temperature to 22°C. Subsequently, the mixture was transferred to 35 µL rRNA removal beads (Ribo‐Zero plant kit), and incubated at room temperature before the beads were removed by a magnetic stand.

### 4.8 DMS‐MaPseq Library Construction

DMS‐MaPseq was performed as described with modification [6, 52]. Briefly, the library was constructed starting with rRNA‐depleted RNA using the Illumina TruSeq Stranded Total RNA Sample Prep Plant kit (Illumina) and Thermostable Group II Intron Reverse Transcriptases (TGIRT) (Ingex). The resulted library was sequenced by PE150 on Novaseq 6000 platform.

### 4.9 TYMV Genome Assemble

TYMV genome was assembled by SPAdes toolkit [53] using DMS‐MaPseq data of non‐DMS treated samples.

### 4.10 DMS‐MaPseq Data Analysis

Raw fastq files were subjected to quality control by trimming the Illumina universal adapter and quality control filter which required a quality score > 25 at the 3’ end by cutadapt [54]. To further improve the sequence quality, “Fastq_quality_filter” from the “Fastx‐toolkit” was used to filter sequences with low quality with the parameters “‐q 25 ‐p 80,” indicating that 80% of the nucleotides had a base call accuracy of more than 99.7% (http://hannonlab.cshl.edu/fastx_toolkit/). Then the high‐quality reads were mapped to TYMV genome using TopHat2 with the parameters “‐N 15 –read‐gap‐length 10 –read‐edit‐dist 15 –max‐insertion‐length 5 –max‐deletion‐length 5 ‐g 3” [55] and only uniquely mapped reads were retained for further analysis.

The DMS‐triggered mismatches were called as described before [56, 57]. Briefly, we used a Python script named “CountMismatch2Bed.py” which only needs a bam file as input. The output file is a bed file including location and mismatch count information. BEDTools [58] was utilized to acquire nucleotide coverage using the “genomecov” subcommand with the parameters “‐d ‐split”. Raw DMS reactivity was determined as the ratio between the mismatch count and the coverage for each nucleotide. The average mismatch ratios of A/C/G/U, were calculated and the results were plotted in a bar plot using the R package “ggpubr” (https://cran.r‐project.org/web/packages/ggpubr/index.html). Plotly R (https://plotly.com/r/) was used to create 3D plots based on DMS reactivities for three biological replicates and we calculated Pearson correlation (R value) for each pair.

For the DMS reactivity profile across the TYMV genome (Figure 1c), we normalized as follows:

A sliding window method was adopted to calculate the DMS mismatch ratios across the entire TYMV genome. The whole TYMV RNA was divided into 10‐nt windows. First, we averaged DMS mismatch ratios of three DMS‐treated replicates within each window. Then the DMS mismatch ratios of non‐DMS treated sample was subtracted. Finally, the mismatch ratios of each window were normalized to the top 5% mismatch ratio. If the normalized DMS mismatch ratio is higher than 1, it was winsorized by setting them to 1.

### 4.11 Analysis of Alternative RSS Using DREEM Algorithm

Three replicates were merged to run DREEM algorithm [20] due to their high reproducibility. The whole TYMV RNA was divided into 100‐nt windows and then we ran the DREEM with the default configuration. For each window, the iteration results with the most cluster numbers were used in the following.

We used the Shannon diversity index to represent the RSS diversity in this study.

$$Shannon\;Index=-\sum_{i=1}^{c}p_{i}lnp_{i}$$

where *c* is the cluster number, which may be equal to 1, 2, 3, 4, or 5. *p_i_* is the percentage of the *i* cluster.

### 4.12 RSS Modeling

Raw DMS mismatch ratios were used in the RSS modeling based on bulk DMS‐MaPseq data by RNAstructure [34] (Figure 1d; Figures S2a and S3). In Figure 1d and Figure S2a, a sliding window (window = 2000 nt, step = 100–200 nt depending on where one folding domain ends) and parameter of “‐md 300 ‐t 295” were used to model the RSS except the last window (5600–6318 nt) using “‐md 100 ‐t 295” due to the shorter window. In the output. ct file, there are normally more than one possible structure can be found. To obtain a more accurate structure, we chose the structure that has the same or similar structure in the overlapping region. In Figure S3, a sliding window (window ≈ 700 nt, step = 100–200 nt depending on where one folding domain ends) and parameter of “‐md 300 ‐t 295” were used to model the RSS. DREEM algorithm normalized the DMS mismatch ratios to the median of the top ten mismatch ratios, capping the reactivity at 1. The normalized DMS mismatch ratios were used to model the RSS after DREEM clustering (Figure 2d,g) with the parameter “‐t 295”. RSS was visualized using StructureEditor of RNAstructure [34] or RNAcanvas [59].

### 4.13 Structural Covariance Analysis

Structural covariance analysis was performed as previously described with modifications. Briefly, we retrieved the sequences of *Tymoviridae* family from plant virus database (http://47.90.94.155/PlantVirusBase/) and removed the duplicate sequences. The sequence and structure model of each fragment were used to generate a *stockholm* file. A covariance model (.cm file) was constructed with *cmbuild* (from *Infernal* package [45]) using the *stockholm* file. The covariance model (.cm file) was calibrated using *cmcalibrate* (from *Infernal*). The homologous aligned sequences were retrieved from the *Tymoviridae* database with *cmsearch* (from *Infermal*). Then the duplicated sequences in the alignment file were removed. The remaining sequences were used to build a new covariance model with *cmbuild*. The new covariance model was used to search homologous sequences. This process was repeated until no new sequences could be added. The final alignment file was used as input in R‐scape [60]. The pairs with E‐value equal or smaller than 0.05 are considered as significant covariation.

### 4.14 Reverse Transcription and Quantitative Real‐Time PCR (RT‐qPCR)

Total RNA was extracted from 0.1 g grounded material powder using TRI reagent. Followed by DNase treatment, the cDNA was synthesized using SuperScript II Reverse Transcriptase (Invitrogen) with random hexamers. qPCR was performed as previously described [56]. Primers are listed in Table S1.

### 4.15 Western Blot Assays

Western blot assays were performed as previously described. The blots were probed with a specific antibody GFP (Sigma–Aldrich, G1544‐100UG). The secondary antibody was an anti‐rabbit IgG(GE Healthcare, NA934). Western blot membranes were developed with ECL+ substrate (Biorad).

### 4.16 Transient Expression in Protoplast and Confocal Assays

The plants for protoplasts extraction were grown and the protoplast were prepared as described previously [61]. Plasmids carrying *P_35S_‐2XYFP‐3’ TLS* and 3’ TLS variants were expressed in the protoplasts. Sixteen hours after transfection, the fluorescence signal was captured and evaluated with Leica SP8 confocal microscope. The excitation wavelength for YFP and chlorophyll autofluorescence was 514 and 633 nm, respectively. The pictures were captured under the same setting including laser intensity and smart gain. At least 30 individual protoplasts were examined for each transfection.

### 4.17 RdRP Complex Purification

RdRP complex purification was performed as previously described with modifications [62, 63]. Briefly, the infected Cabbage on 10 dpi was collected and ground into fine powder. 10 g powder was homogenized in 20 mL buffer A (50 mm Tris‐HCl pH7.4, 7.5 mm MgCl_2_, 0.5 mm EDTA, 20% Glycerol, 2 mm DTT) in a mortar. Centrifuge at 3000 g for 3 min at 4°C. Layer the supernatant on top of 6 mL buffer B (25 mm Tris‐HCl pH7.4, 7.5 mm MgCl_2_, 0.5 mm EDTA, 20% Glycerol, 2 mm DTT). Centrifuge on 16 000 rpm for 30 min at 4°C. The pellet was solubilized in 2 mL buffer B supplied with 5% NP‐40 and 0.75 m KCl for 2 h on ice. 10 µL of Micrococcal Nuclease (NEB, M0247S) was added and a final concentration of 5 mm CaCl2 was supplied to remove endogenous RNA. The solubilized mixture was subjected to 25%–55% glycerol gradient ultracentrifuge (Beckman) at 22 000 rpm for 20 h using SW41 rotor. Take 1 mL fractions from bottom to top. Each fraction was added EGTA at a final concentration of 5 mm. Each fraction was tested for activity and the one with the highest activity was further used in the RdRP assay.

### 4.18 In Vitro Transcription

The full‐length virus vectors were linearized by HindIII and blunt by mung bean nuclease. The purified products were used as templates for in vitro transcription. The in vitro transcription was performed using NEB HiScribe T7 High Yield RNA synthesis kit with spiking of 8 mm final concentration of m^7^G(5’)ppp(5’)G RNA cap analog (NEB) to obtain capped RNA. The DNA template was subsequently digested by DNase. The newly transcribed RNA was purified by RNAClean XP beads.

### 4.19 RNA In Vitro Folding

4 µg RNA was added to 2 µL 5X buffer (50 mm Tris‐HCl pH8.0, 100 mm NaCl, 5 mm EDTA) and the total volume was brought to 10 µL with RNase‐free water. Heat the RNA samples at 95°C for 2 min and then slowly cool down.

### 4.20 RdRP Assay

For in vitro RdRP assay, 25 µL of reaction mixture containing 50 m Tris‐HCl (pH 8.5), 10 m MgCl_2_, 10 mM DTT, 100 µg/mL Actinomycin D, 4 m ATP, 4 m CTP, 4 m GTP, 4 m UTP, 1 µ [α‐^32^P]UTP (3000 Ci/mmol), 10 Unit Super‐In RNase inhibitor, and 1 µL RdRP complex were mixed. Then 4 µg in vitro folded RNA was added to the mixture and incubated at 30°C for 2 h. If provided [α‐^32^P]UTP alone, the A/C/G/UTP were substituted with the same volume of 50 mM Tris‐HCl (pH 8.5). After the reaction, the mixture was purified by phenol‐chloroform and fractionated on 12% urea PAGE gel. The RdRP products were visualized by radiography. The experiments were repeated three times for statistical analysis.

## Author Contributions

J.Z. and X.Z. conceived the project and designed the experiments. J.Z. performed the experiments and analyzed the data. C.L. conducted most of the bioinformatic analysis. X.Y., X.L., S.Z., and T.O. provided experimental support. C.O. constructed part of the vectors. J.Z. wrote the initial draft of the manuscript and X.Z. thoroughly edited the paper.

## Conflicts of Interest

The authors declare no conflicts of interest.

## Supporting information




**Supporting File 1**: advs75614‐sup‐0001‐FigureS1‐S8.docx.

**Supporting File 2**: advs75614‐sup‐0002‐TableS1‐S2.xlsx.

## Data Availability

All data are available in the main text or the supplementary materials. High‐throughput sequencing data generated by this study can be accessed in the Genome Sequence Archive (GSA) under accession code CRA042275. Genetic materials will be available when requested.
