# Supplementary material for: DMS‐MaPseq and DREEM Analyses Implicate the Critical Role of RNA Structural Dynamics in Turnip Yellow Mosaic Virus Pathogenicity
Source: Adv Sci (Weinh). 2026 May 8;13(43):e75614. doi: 10.1002/advs.75614 (PMC13335997; doi:10.1002/advs.75614)
Supplement: Supplementary file 1 — Supporting File 1: advs75614‐sup‐0001‐FigureS1‐S8.docx. [file ADVS-13-e75614-s001.docx]

**Supplemental Figures S1-S8**

Mismatch ratio

145-148

144-145

Sequence length (bp)


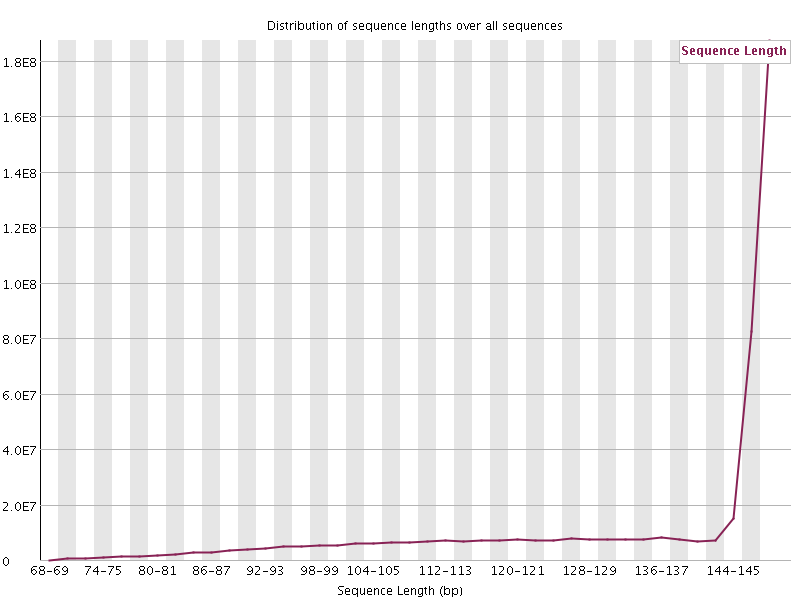

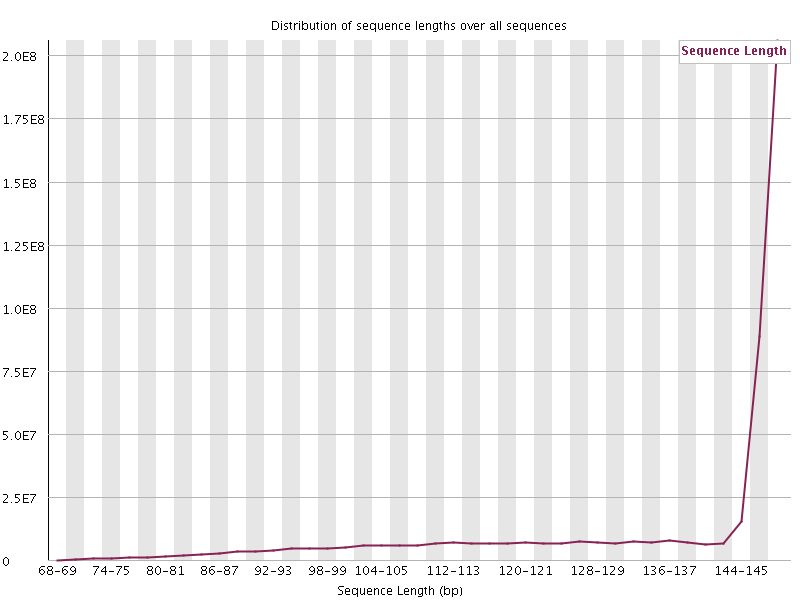

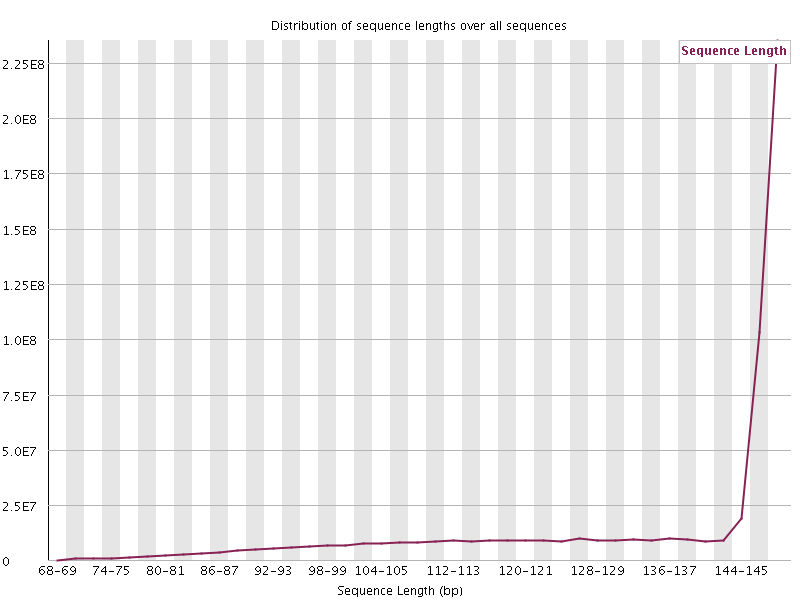


Distribution of sequence lengths over all sequences

Rep 1:

Rep 2:

Rep 3:

68-69

80-81

92-93

104-105

120-121

136-137

1.8E8

1.4E8

1.0E8

6.0E7

2.0E7

0

2.0E8

1.5E8

1.0E8

5.0E7

0

2.0E8

1.5E8

1.0E8

5.0E7

0


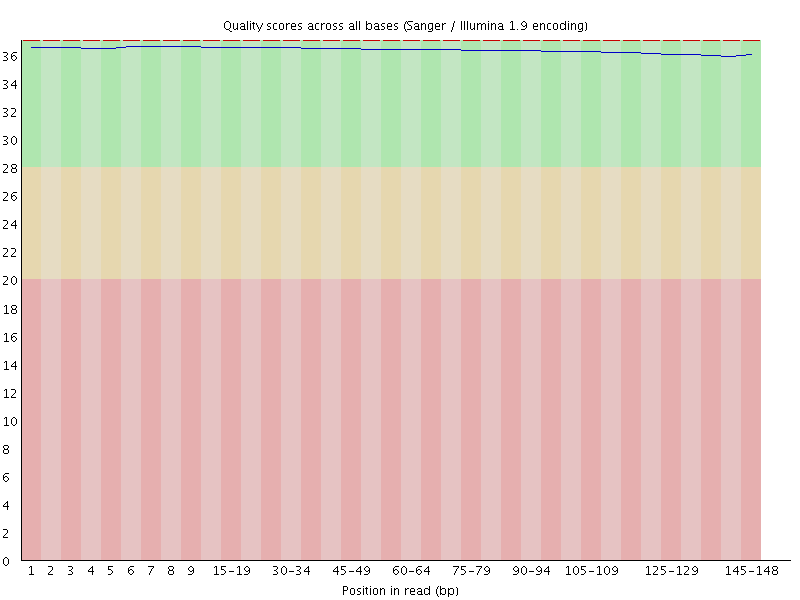

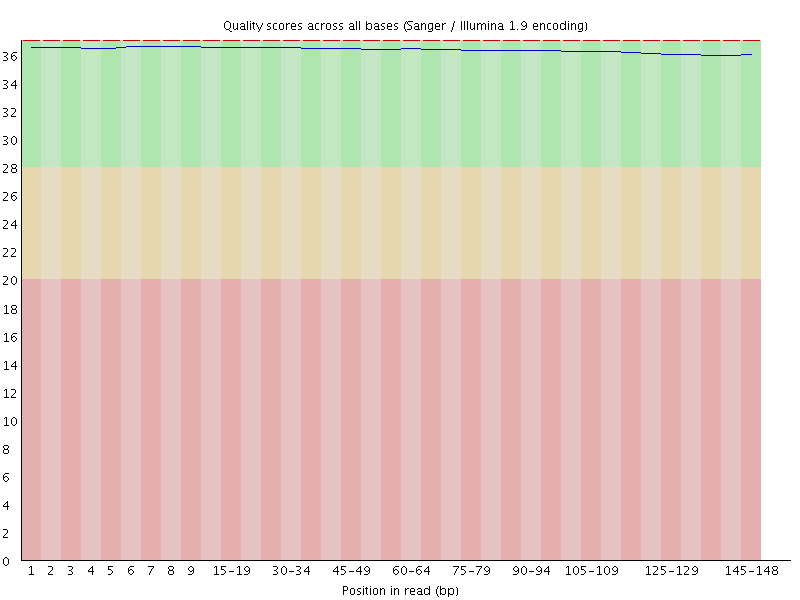

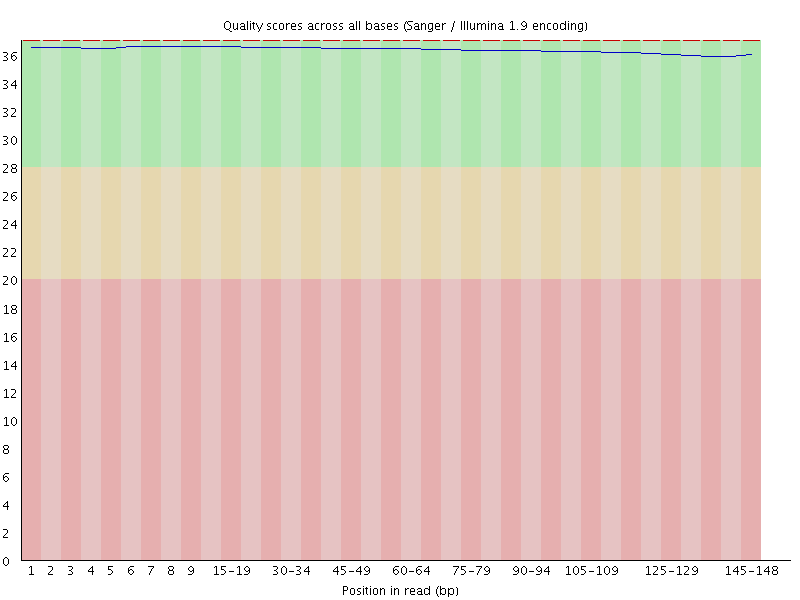


Quality scores across all bases

Rep 1:

Rep 2:

Rep 3:

1

5

15-19

45-49

75-79

105-109

Position in read (bp)

36

32

28

24

20

16

12

8

4

0

36

32

28

24

20

16

12

8

4

0

36

32

28

24

20

16

12

8

4

0


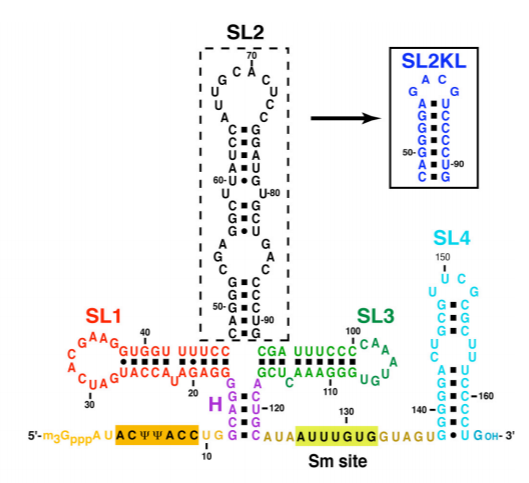


Human:


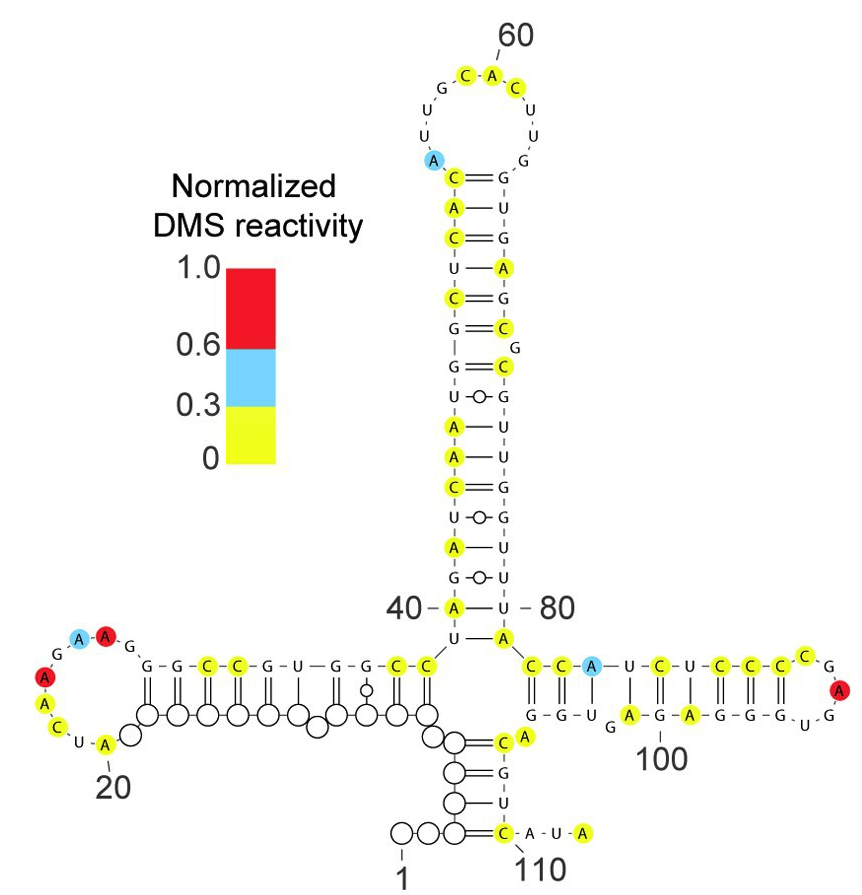


Rice:


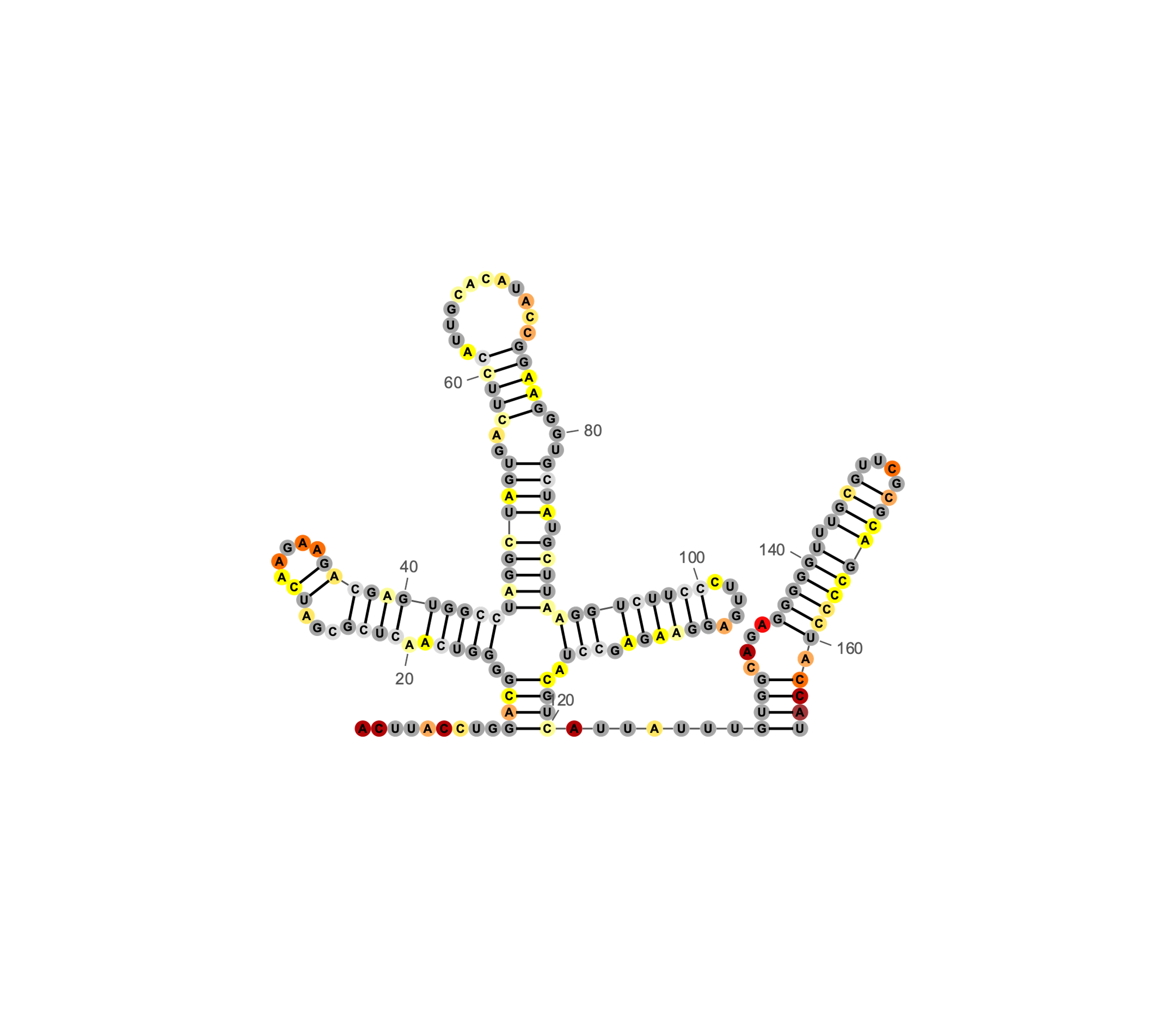


This study:


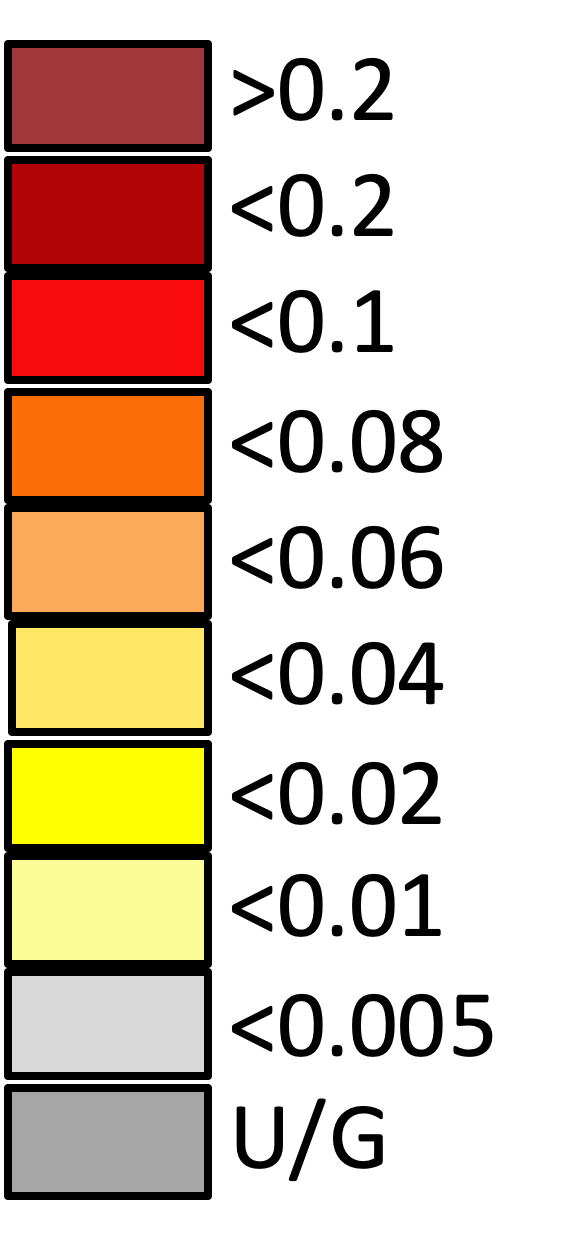

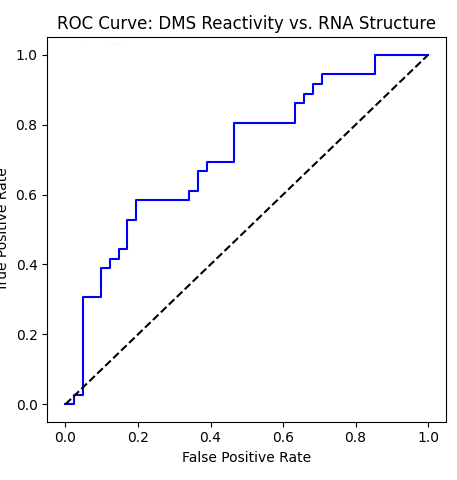


True Positive Rate

False Positive Rate

1.0

0.8

0.6

0.4

0.2

0.0

1.0 0.8 0.6 0.4 0.2 0.0

AUC=0.713

a.

b.

c.

d.

e.

f.

g.

h.


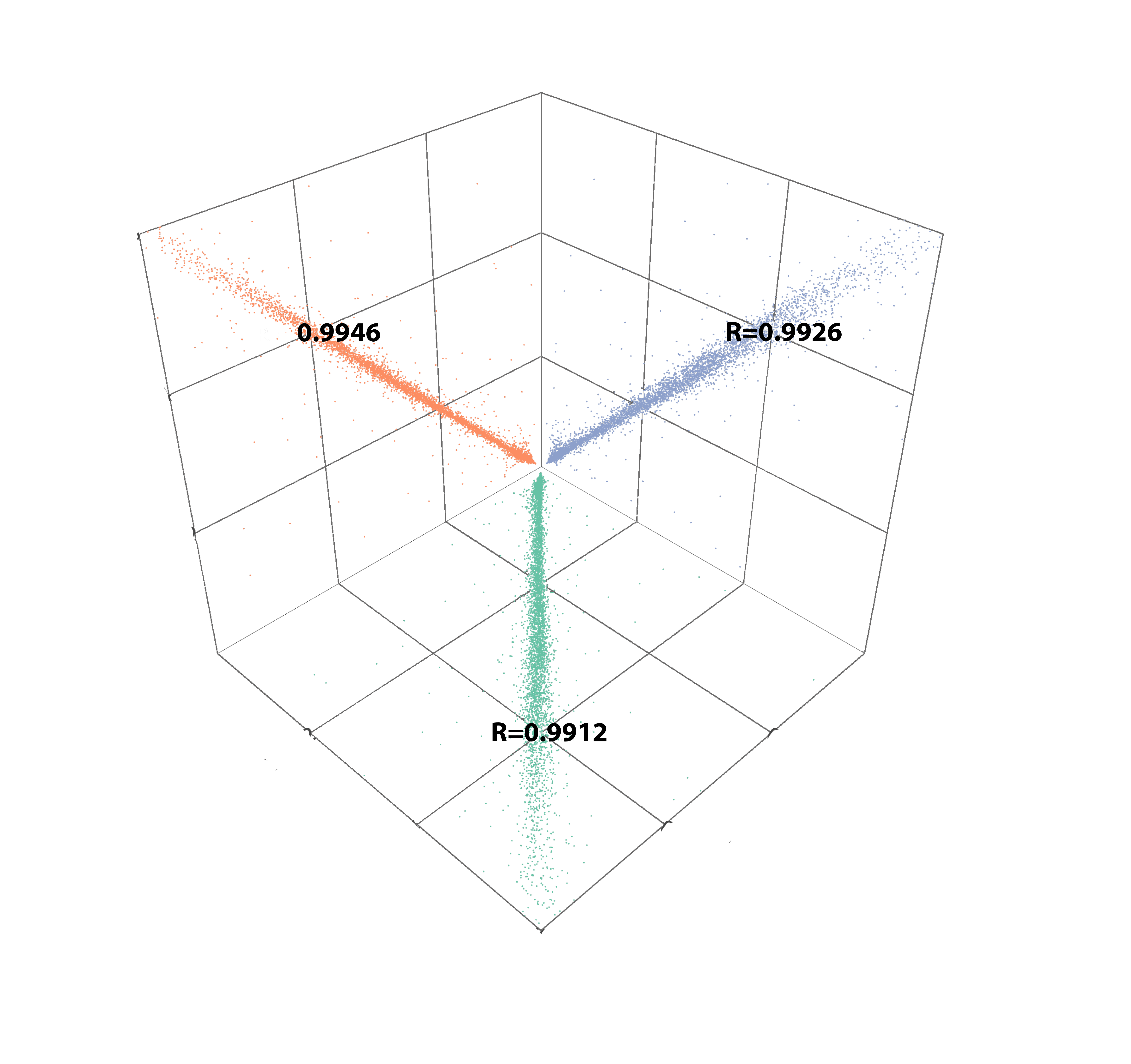


0.06

0.04

0.02

0

0.02

0.04

0.06

0.06

0.04

0.02

0

DMS R3

DMS R2

DMS R1


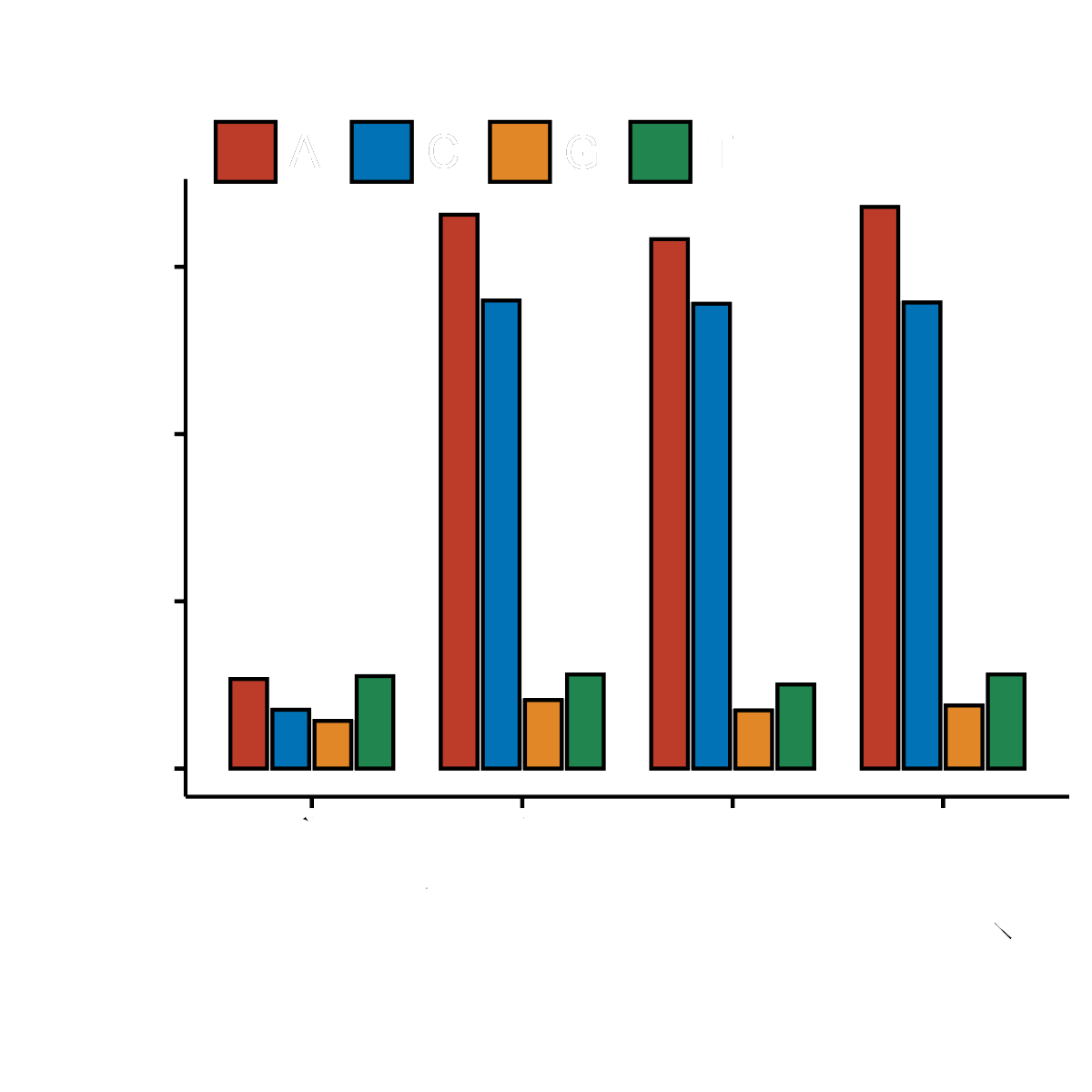


DMS R1

DMS R2

DMS R3

No DMS

A

C

G

U

0.03

0.02

0.01

0.00

Average mismatch ratio

**Figure S1. DMS-MaPseq produces high-quality data for structural analysis.**

**a,** FastQC test reveals per-base sequencing quality scores for three biological replicates. The green, yellow and red colors denote high, medium and low-quality scores.

**b,** FastQC of the distribution of sequence lengths across all reads from three biological replicates indicates the integrity of cDNA libraries under the DMS treatment. Be noted: the Illumina sequencing was performed with the PE150 setting.

**c,** Total mismatch ratio on each nucleotide in DMS-treated and mock-treated samples.

**d,** Pearson’s correlation coefficient analysis shows high reproducibility among different biological replicates for DMS-treated samples.

**e,** RSS of human U1-snRNA that is earlier determined by crystal structure^36^.

**f,** RSS of rice U1-snRNA that is earlier determined by DMS-MaPseq^35^.

**g,** The predicted secondary structure of U1snRNA based on the DMS-MaPseq data in this study. The nucleotides are color-coded according to mismatch ratios.

**h,** ROC curve between U1-snRNA DMS mismatch data and the published human U1-snRNA crystal structure. The U1-snRNA sequences of human and Arabidopsis were aligned via Clustal Omega.




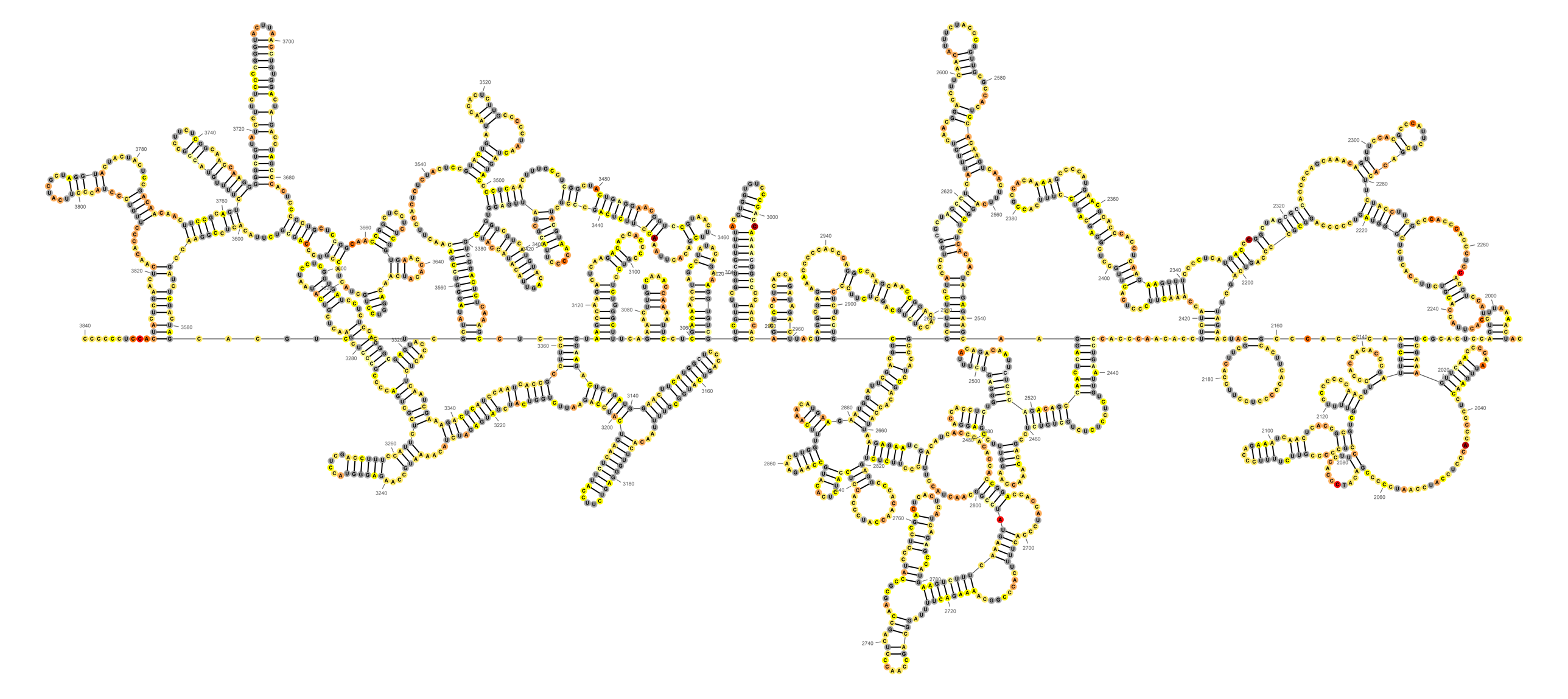



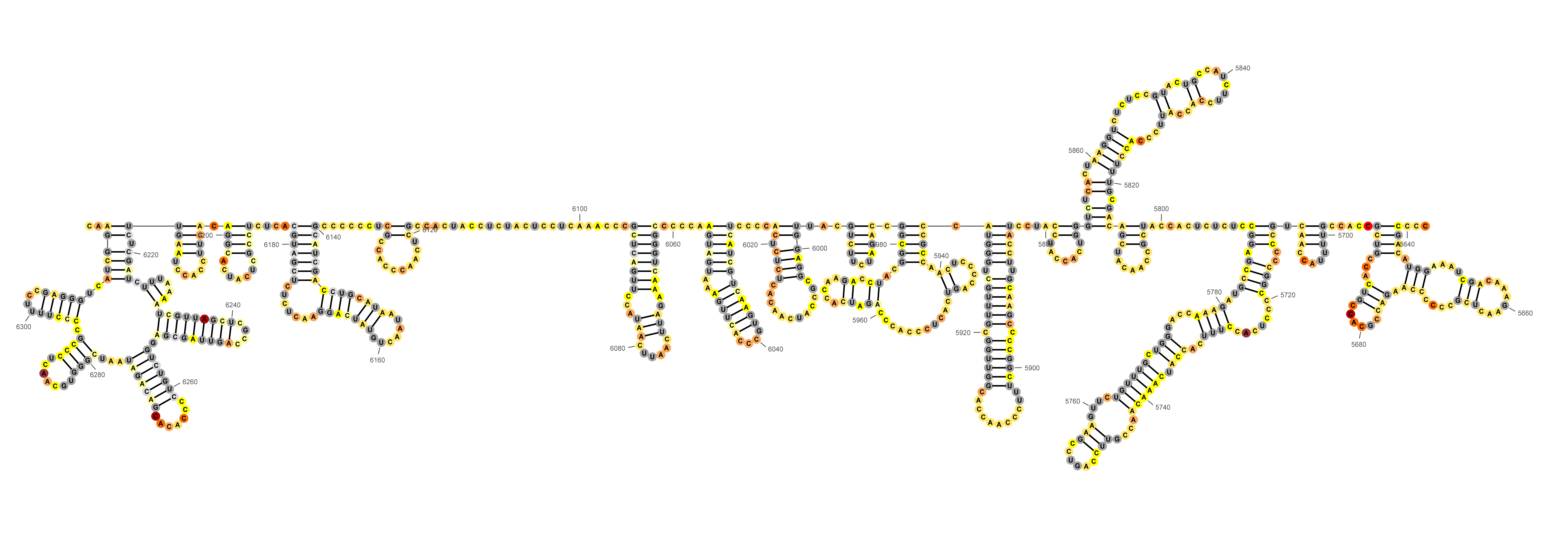

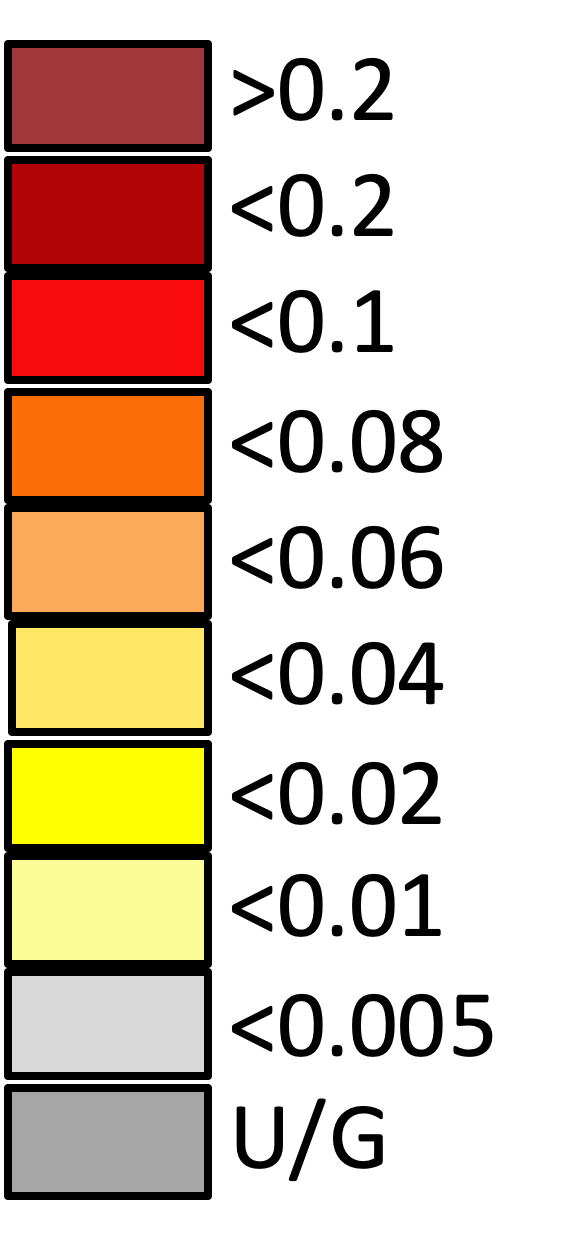


Mismatch ratio

a.

SLs

b.


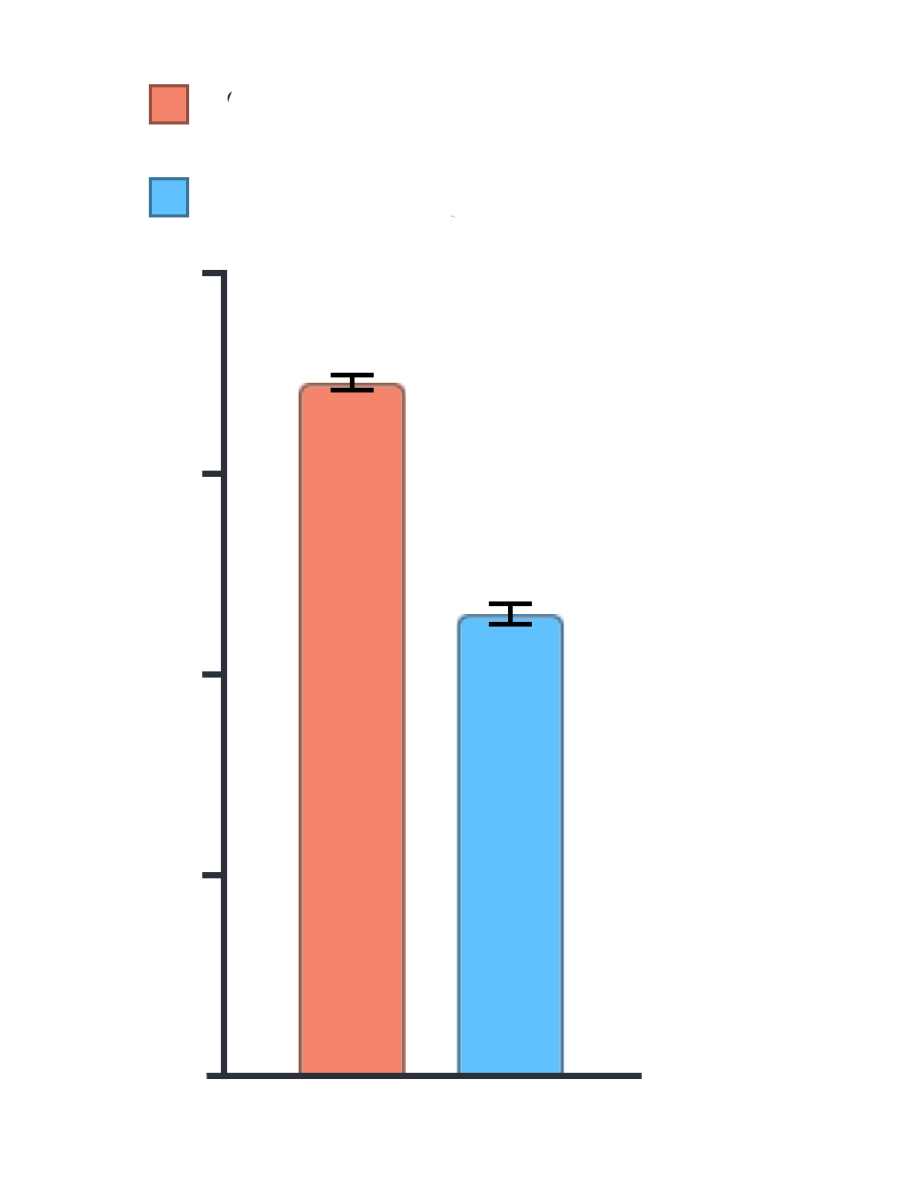


Mismatch ratio

Single-stranded

Double-stranded

0.00

0.01

0.02

0.03

0.04

***

**Figure S2. The RSS landscape of TYMV genome profiled by DMS-MaPseq.**

**a,** DMS-MaPseq of bulk reactivities shows numerous short stem-loop clusters and approximately 21 large complicated RSS loci cross the TYMV genome. The nucleotides are color-coded as the mismatch ratio. Be noted: the pink arrows represent some nucleotides with high DMS signals located in double-stranded regions.

**b,** The average mismatch ratios in single-stranded and double-stranded regions, respectively. The single-stranded and double-stranded regions were defined according to the RSS in (a). The data are presented as mean ± SD from three biologically independent replicates. The asterisks indicate the significance of the differences between single-stranded regions and double-stranded regions (*P < 0.05; **P < 0.01; ***P < 0.001; unpaired two-tailed Student’s t-test).

**
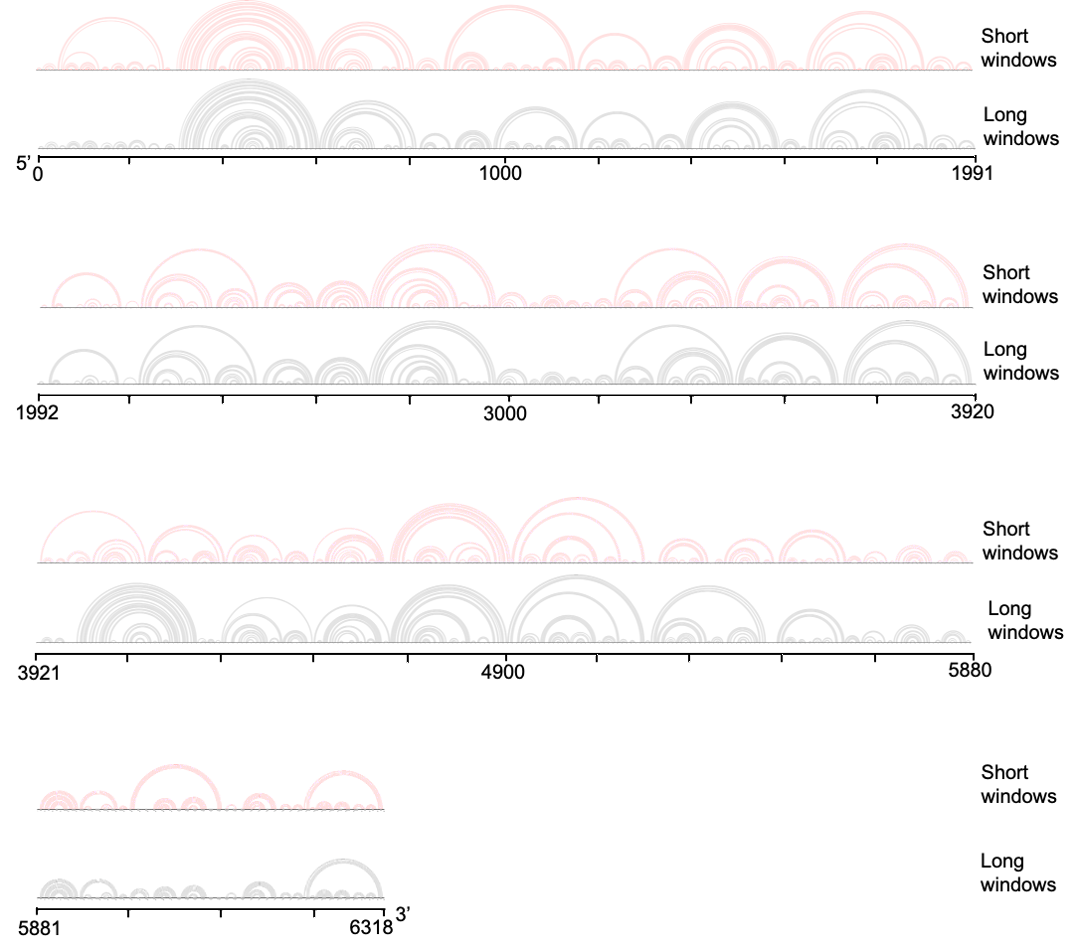
**

**Figure S3. RSS predicted using long and short window sizes suggests window size does not affect RSS prediction.**

The RSS was presented via linear arc diagrams. The two nucleotides connected by an arc indicate that these two nucleotides are base-paired. The pink and blue panels represent the RSS landscapes that were modeled using around 700- and 2000-nt windows, respectively.

a.

b.


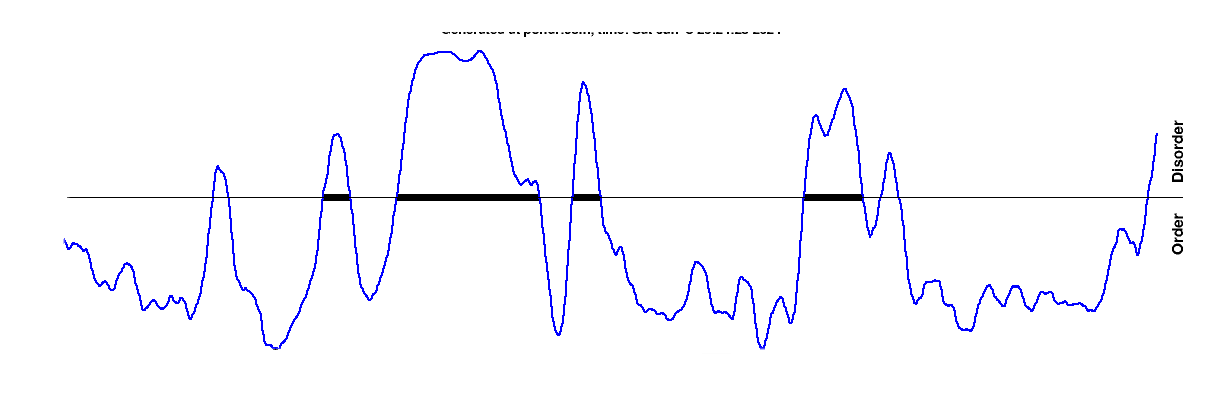


TYMV *206K*

1.0

0.0

Order Disorder

0.8

0.7

0.6

0.5

0.4

0.3

0.2

0.1

0.0

Normalized

mismatch ratio

Viral

methyltransferase

Tymovirus

endopeptidase

RNA helicase

RNA dependent

RNA polymerase


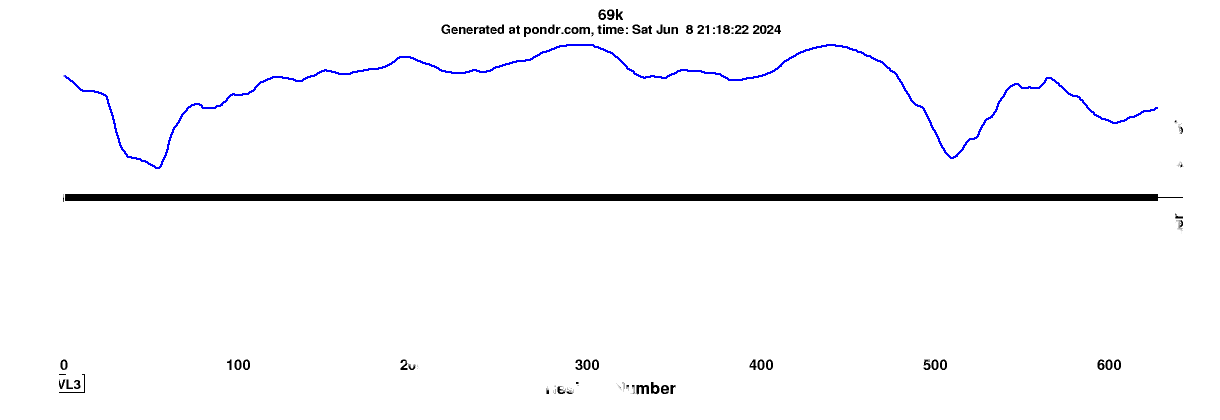


1.0

0.0

Order Disorder

TYMV *69K*

0.8

0.7

0.6

0.5

0.4

0.3

0.2

0.1

0.0

Normalized

mismatch ratio

TYMV 45/70 kD

**Figure S4. The alignment between DMS mismatch ratio and protein folding does not suggest there is any correlation between them in TYMV as observed in HIV.**

a, Top, the schematic of the domains in TYMV 206K protein. Middle, normalized mismatch ratio per nucleotide across the TYMV 206K protein. The data is from merged replicates (n=3). Bottom, predicted disordered regions on 206K protein.

**b,** Top, the schematic of the domains in TYMV 69K protein. Middle, normalized mismatch ratio per nucleotide across the TYMV 69K protein. The data is from merged replicates (n=3). Bottom, predicted disordered regions on 69K protein.


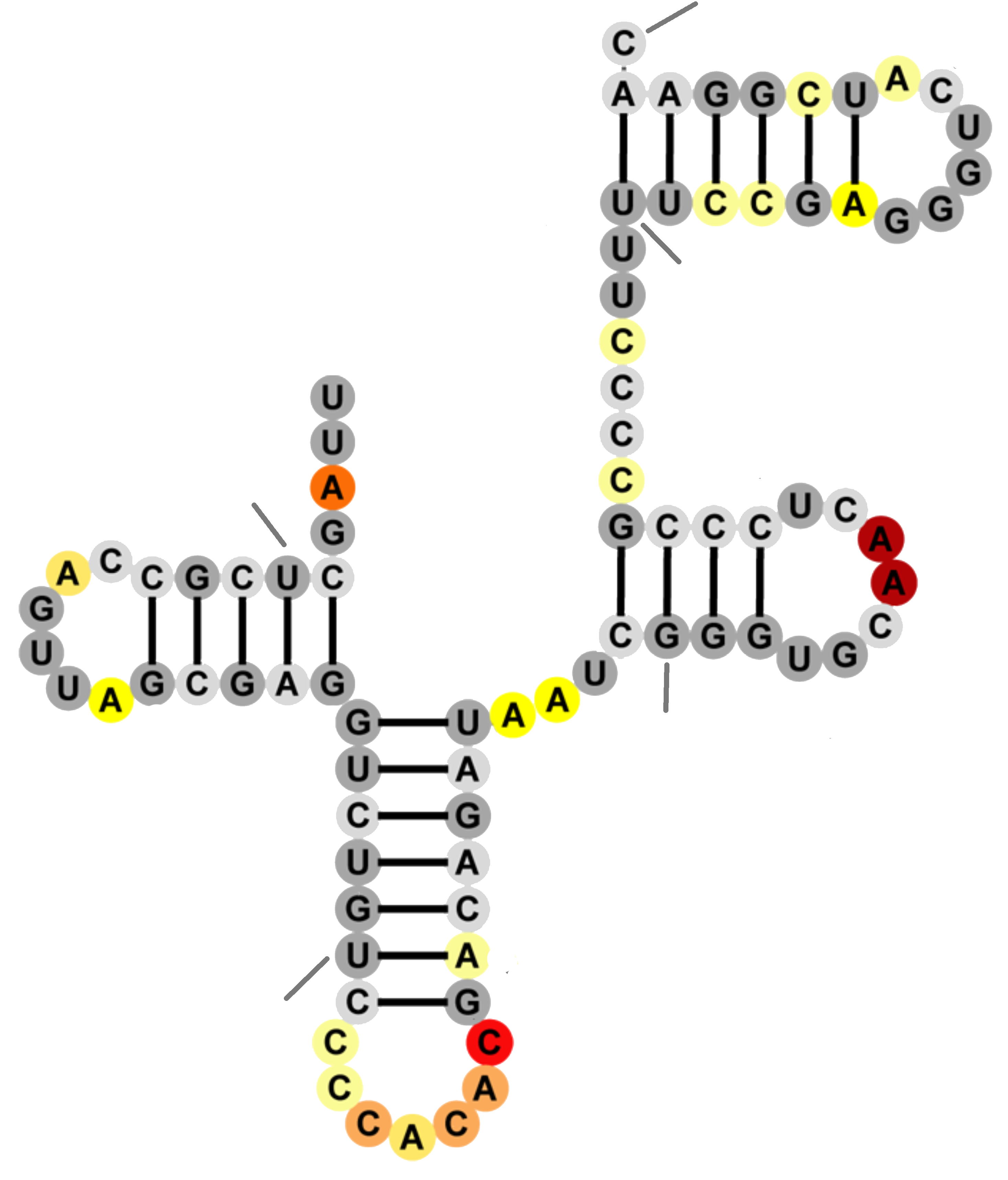


Normalized mismatch ratio


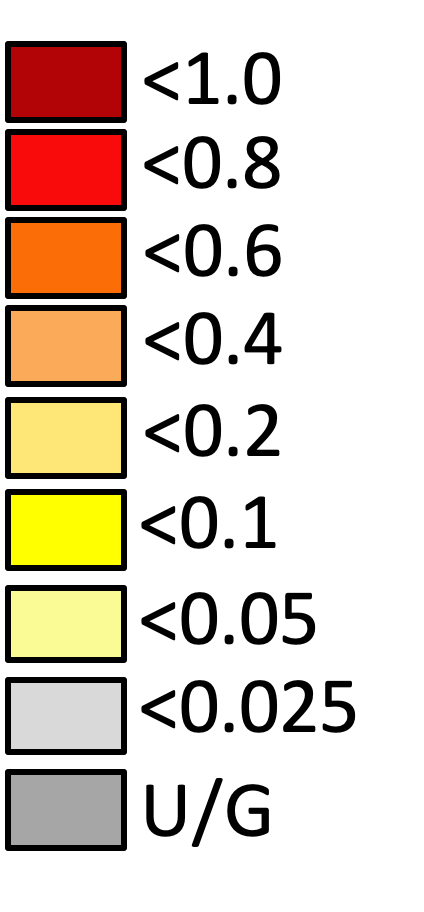


3’ TLS Structure 1

6240

6260

6280

6300

6318

40


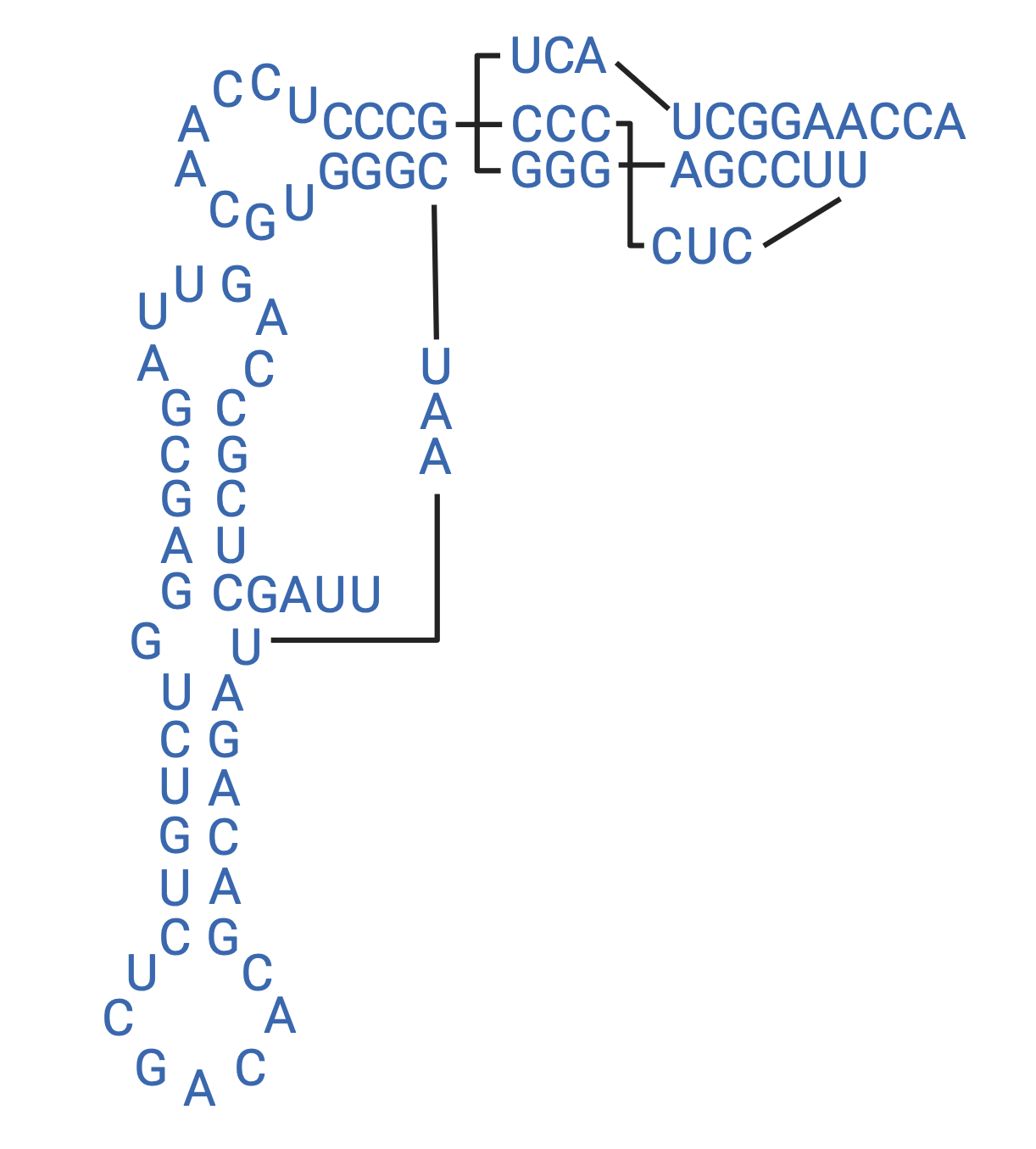


5’

3’

10

20

30

50

60

70

80

a.


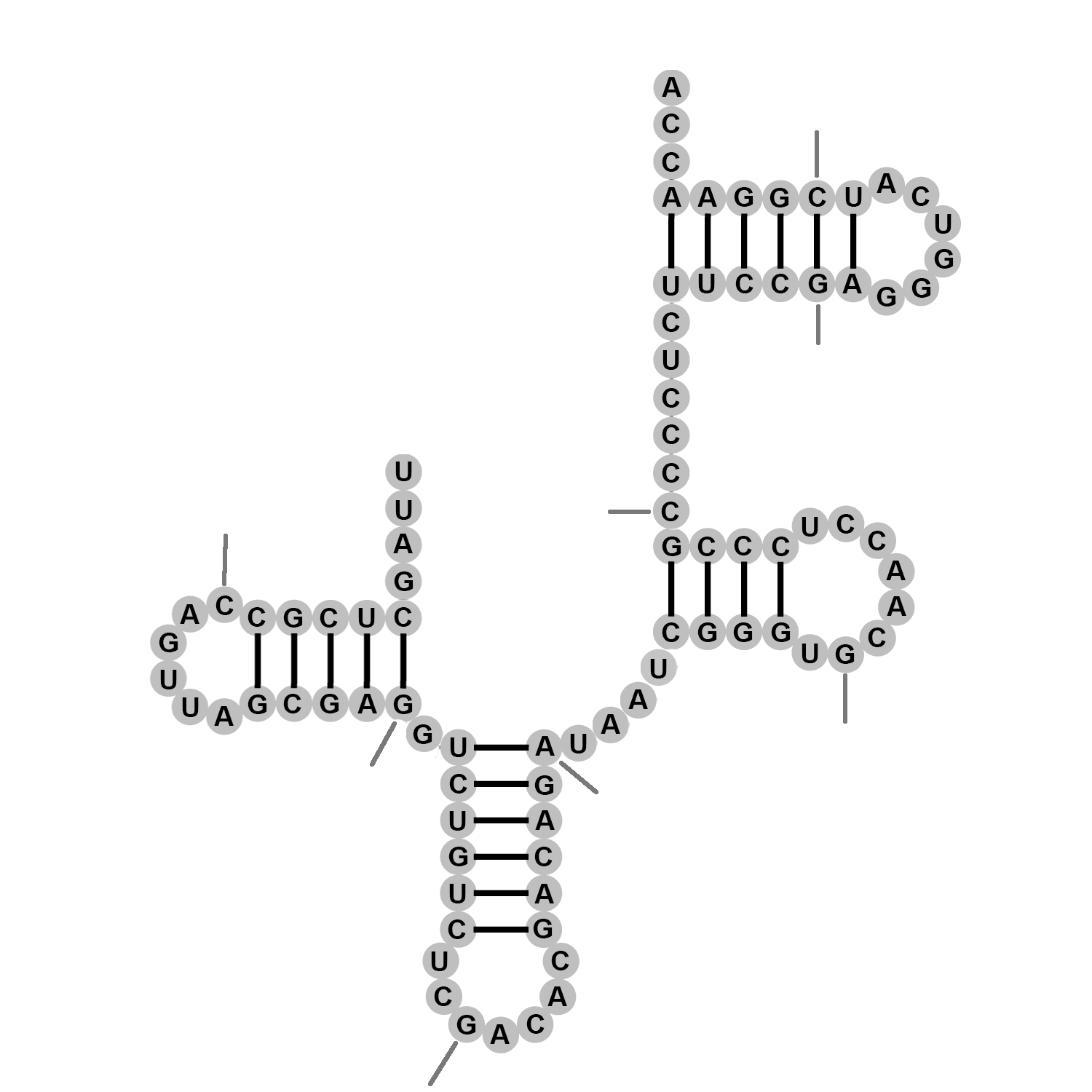


5’

3’

10

20

30

40

50

60

70

80

b.

c.

**Figure S5. Structure #1 of 3’ TLS from DREEM has the same structure as the earlier reported in vitro structure from X-ray crystallography.**

**a,** Reported in vitro 3’ TLS structure from X-ray crystallography^26^ .

**b,** The flattened 2D structure of (a).

**c,** Structure #1 of 3’ TLS from DREEM. The nucleotides are color-coded as normalized mismatch ratio.

**Figure S6. Alternative structures in 3’ TLS are indispensable for viral pathogenicity**

**a-c**, DMS-MaPseq of in vitro transcribed full-length WT and mutant viral transcripts validated that the 3’ TLS of Δstem (a), Δpseudoknot (b) and Δstem&pseu (c) displayed the same RSS signatures as designed. The dashed line on Δstem (a) represents the pseudoknot revealed by the reported in vitro X-ray crystallography and NMR^26,33^. The numbers indicated the relative nucleotide positions on the TYMV genome. The nucleotides are color-coded based on in vitro DMS mismatch ratio.

**d-f,** Pearson correlation of DMS mismatches on the 3’ TLS region (d), upstream region of 3’TLS (e), and whole genome except 3’TLS (f) among WT, Δstem, Δpseudoknot and Δstem&pseu RNA showed that the mutations only changed the RSS of 3’ TLS clearly, but not the RSS of control regions (e and f).

**g,** Pathogenicity comparison among WT virus and the variants with Δstem, Δpseudoknot and Δstem&pseu TYMV RNA. Photographs of plants at 9 dpi (days post-inoculation). Scale bars, 1 cm.

**h,** Pathogenicity comparison among WT, and its variants with Δstem, Δpseudoknot and Δstem&pseu TYMV RNA with a spike of 10% WT. Photographs of plants at 9 dpi (days post-inoculation). Scale bars, 1 cm.

qRT-PCR

Normalized *YFP*

RNA level

8

6

4

2

0

Ratio

(WB/ YFP)


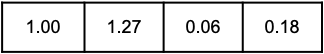


3TLS-WT

3TLS-Δstem

3TLS-Δpseu

3TLS-Δstem&pseu


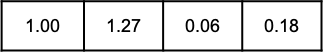


Ratio

(WB/ YFP)

3TLS-WT

3TLS-Δstem

3TLS-Δpseu

3TLS-Δstem&pseu

a.

b.

50


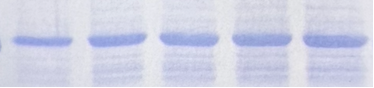


3TLS-WT

3TLS-Δstem

3TLS-Δpseu

3TLS-Δstem&pseu

No transfect


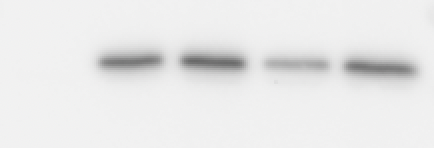


1.00 1.20 0.59 1.11

50

75

KDa

YFP

Rubisco

50


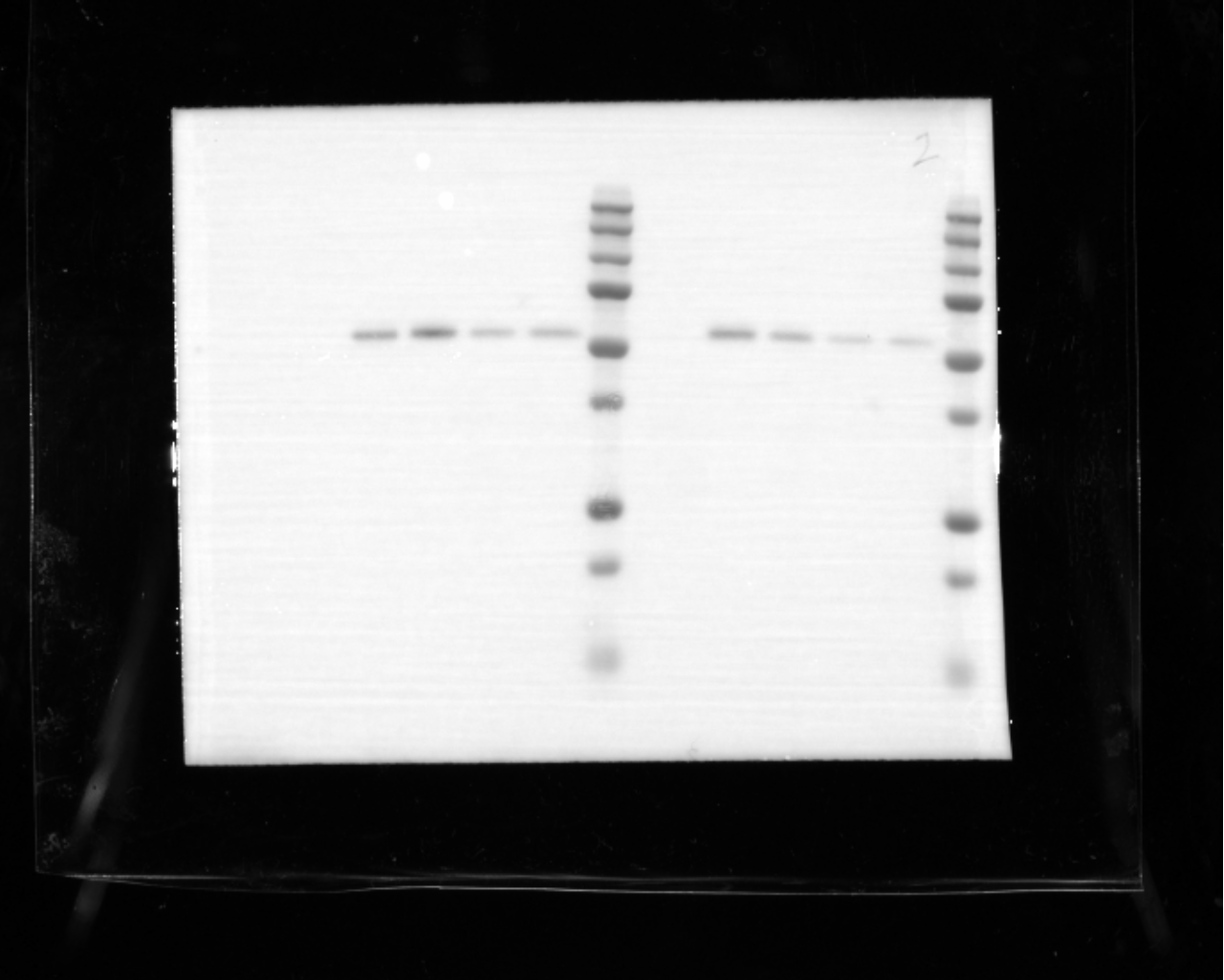


1.00 1.46 0.58 0.79

50

75

KDa

YFP

3TLS-WT

3TLS-Δstem

3TLS-Δpseu

3TLS-Δstem&pseu

No transfect

Rubisco


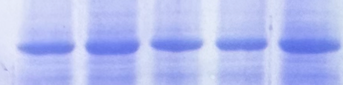


qRT-PCR

5

4

3

2

1

0

Normalized *YFP*

RNA level

c.

d.

1.00

1.00

1.61

0.96

7.05

1.43

3.95

2.51

**Figure S7. Additional repeats show that the pseudoknot structure in 3’TLS facilitates viral translation.**

**a, b,** The other two replicates of Fig. 5d. Western blot assays show YFP protein levels in the protoplasts. The signals were quantified by image J where WT was arbitrarily assigned a value of 1. Coomassie blue staining of Rubisco serves as a loading control.

**c,d,** The other two replicates of Fig. 5e. qRT-PCR assay shows RNA levels of *YFP* in the protoplasts. The *YFP* and *Hygromycin* RNA levels were first normalized to the internal control *UBC10*, and then the *YFP* RNA level was normalized to the *Hygromycin* RNA level, which was co-expressed in the same plasmid with *YFP*. The *YFP*/*Hyg* ratio of the reporter fused with WT 3’ TLS was arbitrarily assigned a value of 1. The protein levels detected from the western blot was then normalized to the *YFP* RNA levels, and the ratios were labeled at the bottom. The protoplast transfection experiments were independently repeated three times, with consistent results.

a.

b.


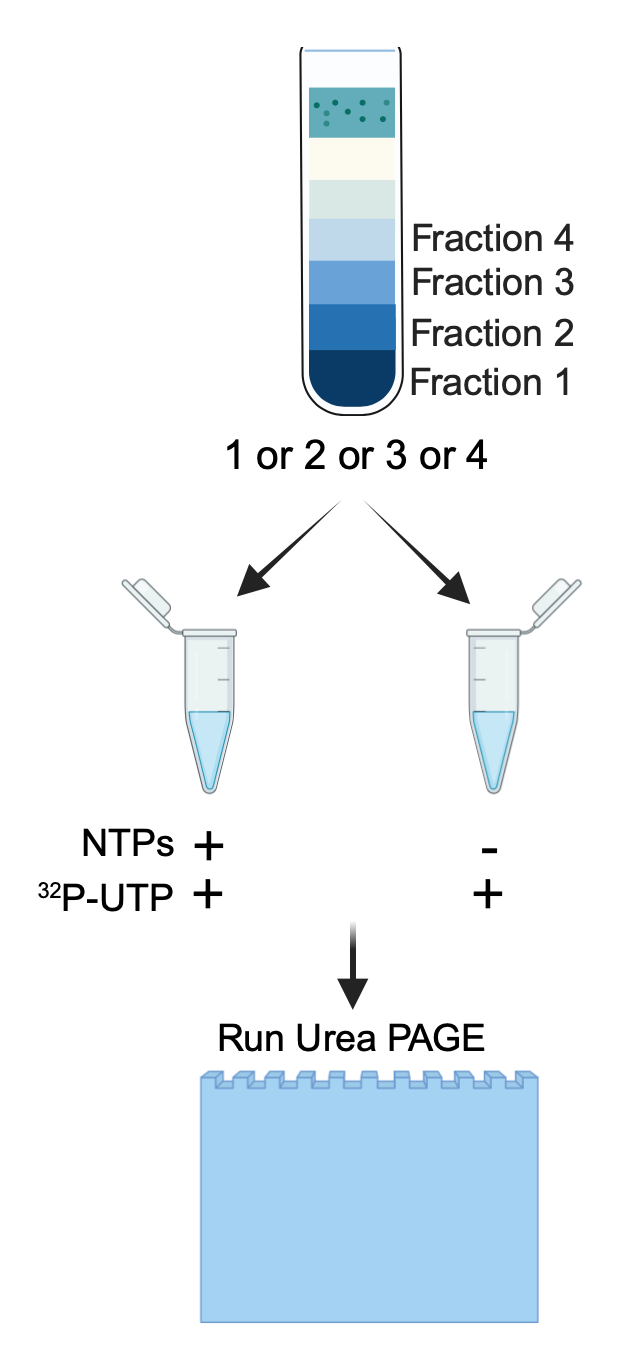


NTPs+^32^P-UTP

Frac

#1

#2

#3

#4

#1

#2

#3

#4

Replicate #1


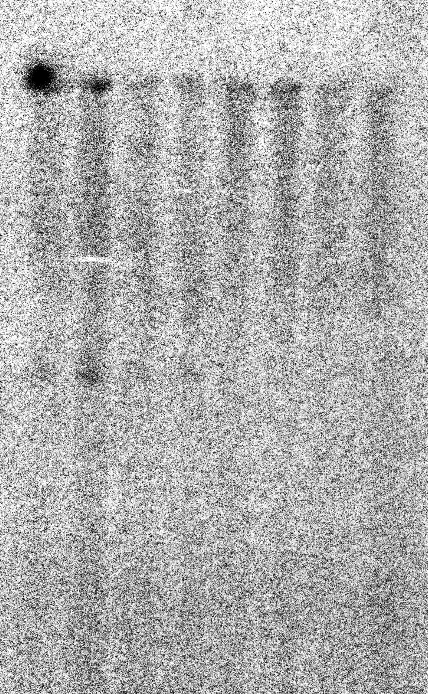


1.00

0.52

0.31

0.30

0.37

0.38

0.30

0.29

NTPs+^32^P-UTP

#1

^32^P-UTP only

#2

#3

#4

Replicate #2


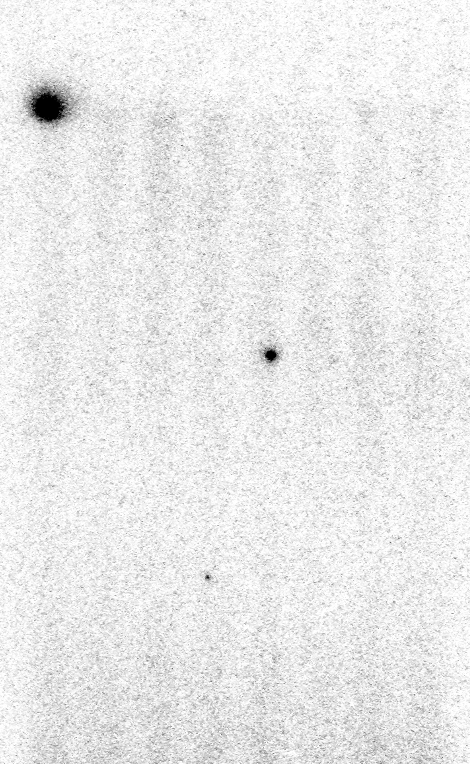


#1

#2

#3

#4

1.00

0.17

0.15

0.11

0.08

0.06

0.09

0.09

^32^P-UTP only

**Figure S8. The purified RdRP complex exhibited predominant RNA-dependent RNA polymerase activity with limited terminal nucleotide transferase activity.**

**a,** Schematic of experimental design to assess RNA-dependent RNA polymerase activity and terminal transferase activity. Following the RdRP complex isolation, different fractions (Fraction #1-4) from the glycerol gradient were collected and tested for RdRP activity.

**b,** Assessment of RdRP and terminal nucleotide transferase activity across glycerol gradient fractions. Fraction #1-4 from glycerol gradient were collected and tested for RdRP activity. Parallel RdRP assays were performed using either all four NTPs (including ^32^P-UTP) or ^32^P-UTP alone. Reaction products were resolved on 12% urea PAGE gel. Fraction #1 exhibited RdRP activity with barely detectable terminal nucleotide transferase activity in our reaction system. Other fractions barely showed either RdRP activity or terminal nucleotide transferase activity.
